# TREM2^hi^ resident macrophages protect the septic heart by maintaining cardiomyocyte homeostasis

**DOI:** 10.1038/s42255-022-00715-5

**Published:** 2023-01-12

**Authors:** Kai Zhang, Yang Wang, Shiyu Chen, Jiali Mao, Yue Jin, Hui Ye, Yan Zhang, Xiwang Liu, Chenchen Gong, Xuejun Cheng, Xiaoli Huang, Andreas Hoeft, Qixing Chen, Xuekun Li, Xiangming Fang

**Affiliations:** 1 Department of Anesthesiology and Intensive Care, The First Affiliated Hospital, Zhejiang University School of Medicine, Hangzhou, China; 2 Department of Critical Care Medicine, Affiliated Hangzhou First People’s Hospital, Zhejiang University School of Medicine, Hangzhou, China; 3 The Children’s Hospital, National Clinical Research Center for Child Health, Zhejiang University School of Medicine, Hangzhou, China; 4 Department of Anesthesiology and Intensive Care Medicine, University Hospital of Bonn, Bonn, Germany; 5 The Institute of Translational Medicine, School of Medicine, Zhejiang University, Hangzhou, China

**Keywords:** Bacterial infection, Bacterial infection, Metabolism, Cardiovascular biology

## Abstract

Sepsis-induced cardiomyopathy (SICM) is common in septic patients with a high mortality and is characterized by an abnormal immune response. Owing to cellular heterogeneity, understanding the roles of immune cell subsets in SICM has been challenging. Here we identify a unique subpopulation of cardiac-resident macrophages termed CD163^+^RETNLA^+^ (Mac1), which undergoes self-renewal during sepsis and can be targeted to prevent SICM. By combining single-cell RNA sequencing with fate mapping in a mouse model of sepsis, we demonstrate that the Mac1 subpopulation has distinct transcriptomic signatures enriched in endocytosis and displays high expression of TREM2 (TREM2^hi^). TREM2^hi^ Mac1 cells actively scavenge cardiomyocyte-ejected dysfunctional mitochondria. *Trem2* deficiency in macrophages impairs the self-renewal capability of the Mac1 subpopulation and consequently results in defective elimination of damaged mitochondria, excessive inflammatory response in cardiac tissue, exacerbated cardiac dysfunction and decreased survival. Notably, intrapericardial administration of TREM2^hi^ Mac1 cells prevents SICM. Our findings suggest that the modulation of TREM2^hi^ Mac1 cells could serve as a therapeutic strategy for SICM.

## Abstract (web summary)

Zhang et al. identify a unique population of cardiac-resident macrophages that could be a potential therapeutic strategy for sepsis-induced cardiomyopathy.

## Main

Sepsis affects approximately 49 million individuals and causes over 11 million fatalities worldwide annually^1,2^. The majority of patients with sepsis display aberrant cardiac function, usually termed SICM^3^. As an important contributor to organ dysfunction, SICM is characterized by a reduced ejection fraction (EF) and is associated with high mortality in patients. Of note, transiently reversible myocardial dysfunction is also observed in SICM, suggesting that cardiac homeostasis can be restored after the septic stress^4–6^; however, the mechanisms facilitating heart rehabilitation during sepsis have not been fully elucidated.

Immune cells play vital roles in regulating tissue homeostasis following cardiac injury^7^. In heart tissue, macrophages are dominant immune cells, which are highly heterogeneous and diversified in lineage^8–10^. During the past decade, bone-marrow-derived macrophages (circulating) have been regarded as the only primary phagocytes, which are involved in, for example, cardiac tissue healing and homeostasis^11^; however, emerging evidence indicates that cardiac-resident macrophages (CRMs) play fundamental roles in the clearance of necrotic cells and debris, promotion of angiogenesis, restriction of inflammation and tissue remodeling during the cardiac injury process, which are independent of circulating macrophages^10,12,13^. Very recently, a study showed that CRMs removed the decaying mitochondria ejected by cardiomyocytes to maintain heart health^14^.

A septic heart has a high energy requirement under stress, including elevated body temperature, hypoxia, tachycardia and systemic cytokine storm. In line with this pathological change, cardiac mitochondrial dysfunction has been demonstrated in many animal studies^15,16^. Clinically, mitochondrial dysfunction correlates directly with poor sepsis outcomes^17,18^. Therefore, the maintenance of cardiac mitochondrial homeostasis is essential for the recovery of SICM; however, how immune cells serve as a manipulator of mitochondrial quality control in the septic heart remains completely unknown.

In the present study, by combining single-cell RNA sequencing (scRNA-seq) with fate-mapping techniques, we profiled dynamic changes of cardiac immune cells, especially cardiac macrophages, in the murine septic heart. We identified a CD163^+^RETNLA^+^ Mac1 subpopulation with self-renewal ability that was critical for supporting the cardiac function under septic stress. Deep-phenotypic and functional analysis revealed that the Mac1 subpopulation was characterized by endocytic transcriptomic signatures and by high TREM2 expression. In addition, we observed that the TREM2^hi^ macrophage subpopulation actively scavenged cardiomyocyte-ejected mitochondria and therefore promoted cardiac homeostasis in sepsis. *Trem2* ablation in macrophages impaired the ability of Mac1 cell self-renewal, which resulted in the accumulation of dysfunctional mitochondria in the extracellular space of myocardium and exacerbated cardiac function following sepsis. Finally, intrapericardial administration of TREM2^hi^ Mac1 cells improved cardiac function and prevented SICM. These findings provide a perspective for the development of TREM2-targeted immunotherapies of SICM.

## Results

### Single-cell characterization of immune cells in septic heart

**Extended Data Fig. 1:**
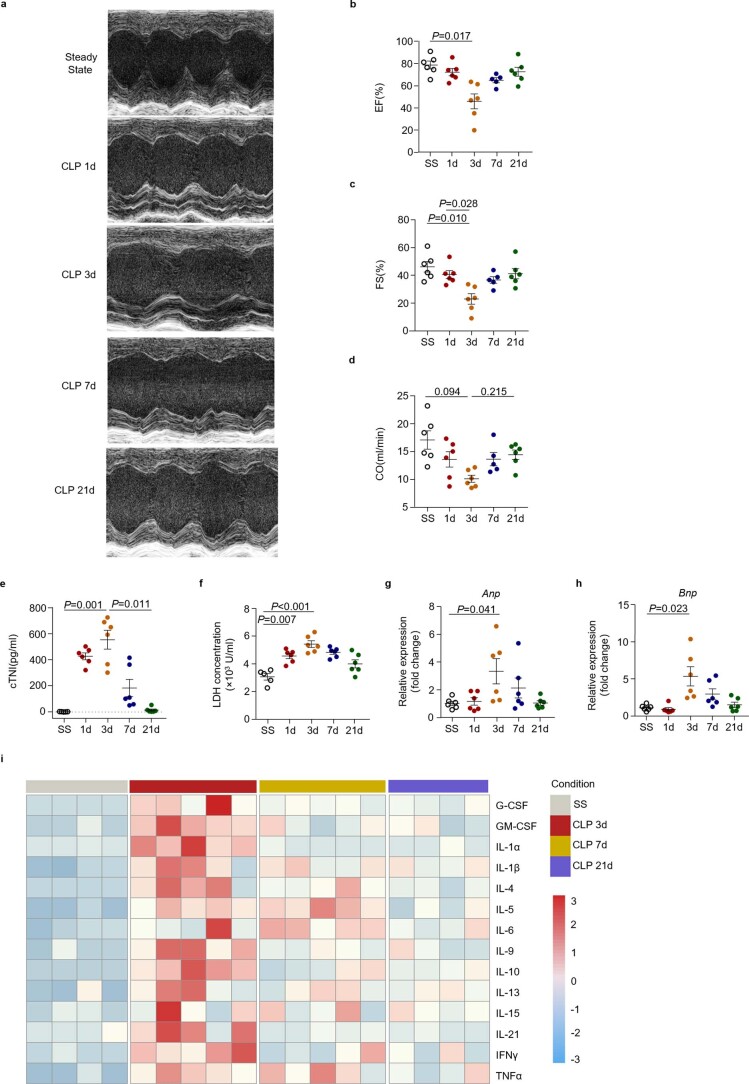
The dynamic changes of cardiac function, injury biomarkers and inflammatory cytokines in SICM, related to Fig. 1. a-d, Representative echocardiography images (a) and EF% (b), FS% (c), and CO (d) measured by echocardiography in Wild-type (WT) mice at steady state (SS), 1, 3, 7, and 21 days after CLP (SS, n = 6 mice; CLP 3 d, n = 6 mice; CLP 7 d, n = 5 mice; CLP 21 d, n = 6 mice). e,f, Graphs showing the levels of cTnI (e) and LDH (f) in the serum. g,h, Graphs showing mRNA levels of *Anp* (g), *Bnp* (h) in the heart tissues; n = 5-6 (e,f,g,h) mice for each treatment. i, Heat map showing the scaled expression of 14 selected cytokines (row) across 22 heart tissue lysate samples (column), colored by different conditions. In b-h, each symbol represents one animal and data are presented as mean ± SEM. Two-sided P values were determined by one-way ANOVA followed by Games-Howell’s (b-d,f) or Tukey’s (g) multiple comparisons test, Kruskal–Wallis with Dunn’s multiple comparison test (e,h). Exact P values are shown. Results represent four independent experiments (b-h). Source data

To explore the mechanisms of reversible myocardial dysfunction during septic stress, we established the septic murine model (cecal ligation and puncture (CLP) model) and profiled the septic heart by echocardiography and cardiac injury biomarkers. As expected, cardiac function deteriorated steadily upon sepsis progression and became significantly worse 3 d after CLP. Subsequently, cardiac function recovered gradually (Extended Data Fig. 1a–d). Congruently, cardiac injury parameters (lactate dehydrogenase (LDH), troponin I, *Anp* and *Bnp*) also showed comparable dynamic changes (Extended Data Fig. 1e–h). Owing to the crucial role of inflammation in the pathology of SICM, cardiac inflammatory markers were measured by a multiplex protein array. The levels of granulocyte colony-stimulating factor, granulocyte-macrophage colony-stimulating factor, interleukin (IL)-4, IL-1α, IL-9, interferon (IFN)-γ, IL-1β, IL-21 and IL-10 in the heart reached a peak 3 d after CLP and then decreased slowly as time progressed (Extended Data Fig. 1i). These results indicate that the dynamic changes of cardiac function during sepsis progression are consistent with the inflammatory environment of cardiac tissue.

**Fig. 1:**
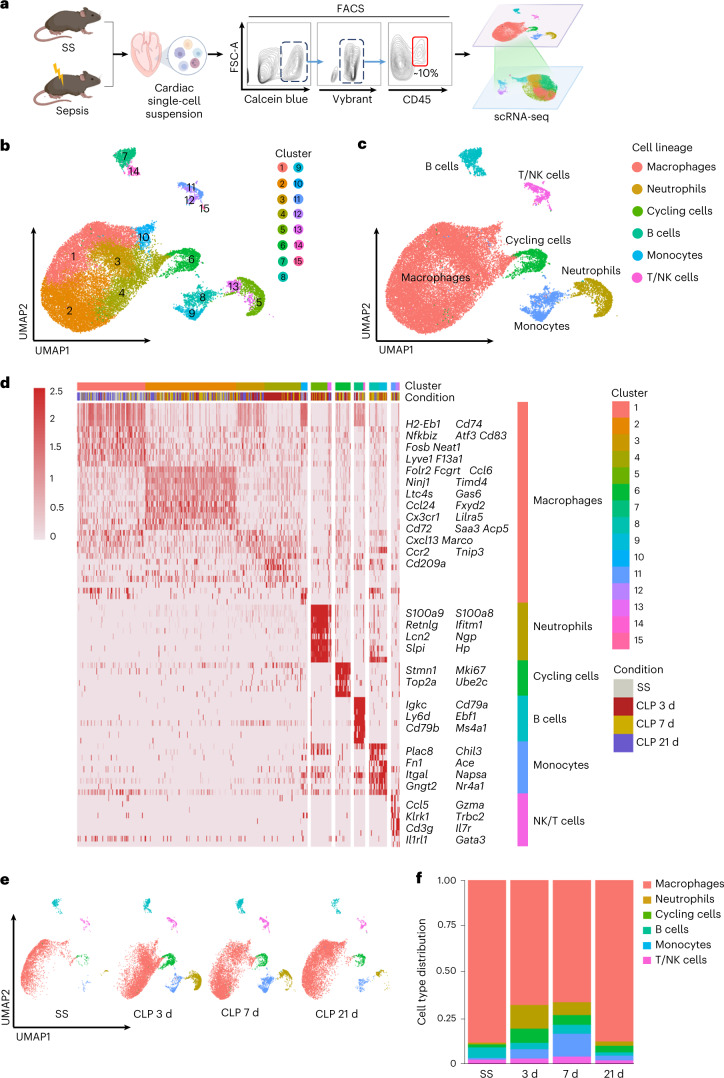
Single-cell atlas of cardiac immune cells during sepsis progression. **a**, Schematic diagram showing scRNA-seq pipeline of murine cardiac immune cells during sepsis progression. SS, steady state. **b**, Uniform Manifold Approximation and Projection (UMAP) plots of 29,537 cardiac immune cells allocated into 15 clusters from the hearts of 14 WT mice at SS, 3, 7 and 21 d after CLP. Cardiac cells from 3–4 mice were mixed as one sample. **c**, UMAP plots of cardiac immune cells colored by cell type. **d**, Heat map showing selected cluster marker genes (top, color-coded by cluster and condition) with exemplar genes and cell-type annotation labeled. **e**, UMAP plots of cardiac immune cells from WT mice in different time points of sepsis, annotated by cell type as in **c**. Each time point is contributed by either three (SS or 21 d) or four mice (3 or 7 d). **f**, Bar plots showing the distribution of immune cell types from WT mice at different time points of sepsis (Extended Data Figs. 1 and 2). Source data

**Extended Data Fig. 2:**
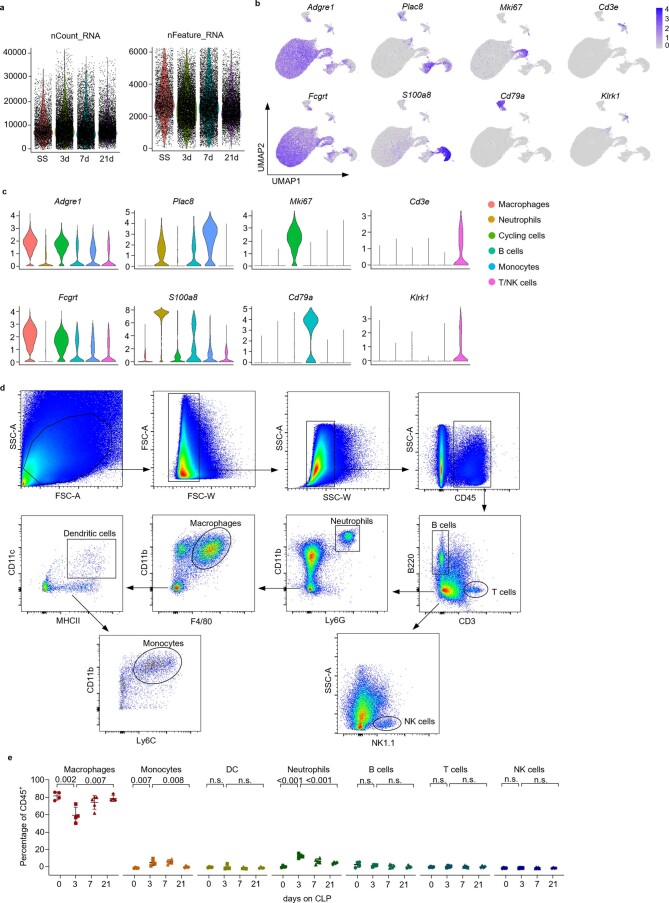
Single-cell characterization and flow cytometric analysis of the cardiac immune cells during sepsis progression, related to Fig. 1. a, Violin plots of average unique molecular identifier (UMI) (left) and gene (right) numbers of scRNA-seq at different stages of sepsis. Each dot represents a cell. b, UMAP plots showing the expression of selected marker genes for the defined cell types in Fig. 1c. c, Violin plots showing the expression of selected marker genes in all defined cell types corresponding to Fig. 1c. d, Gating strategy of 10-color flow cytometry for identifying various types of immune cells in murine hearts. e, Graph showing percentages of cardiac immune cells during sepsis progression (n = 4 - 5 mice for each treatment). Each symbol represents one animal in e. Data are presented as mean ± SEM. Two-sided P values were determined by one-way ANOVA followed by Tukey’s multiple comparisons test, n.s., not significant. In e, experiments were performed four times. Source data

Next, we characterized the types and states of immune cells involved in SICM using scRNA-seq. Metabolically active nucleated CD45^+^ cells were separated by fluorescence-activated cell sorting (FACS) from 14 mice at different time points. A total of 29,537 quality-control-positive cells were selected for further analysis, which generated on average about 9,362 mapped reads and 2,718 genes per cell (Fig. 1a and Extended Data Fig. 2a). As expected, unbiased clustering analysis revealed multiple clusters of cardiac immune cells (Fig. 1b), which included macrophages (*Adgre1* and *Fcgrt*), monocytes (*Plac8* and *Chil3*), neutrophils (*S100a8/a9*), natural killer (NK)/T cells (*Cd3e, Klrk1* and *Il7r*), B cells (*Igkc* and *Cd79a*) and cycling cells (*Mki67* and *Stmn*1) (Fig. 1c,d and Extended Data Fig. 2b,c). Among them, macrophages were the most abundant cells with a rich set of subclusters in the heart. By comparing changes of the immune cells at each time point, we found that the percentages of macrophages slightly decreased, whereas the heterogeneity of macrophage subclusters was more substantial after CLP. Meanwhile, large numbers of neutrophils and monocytes were recruited into the myocardium after sepsis (Fig. 1e,f). In addition to the findings from scRNA-seq, flow cytometry analysis confirmed the parallel changes in cardiac immune cells during sepsis progression (Extended Data Fig. 2d,e).

### Mac1 is associated with restoration of cardiac function

**Fig. 2:**
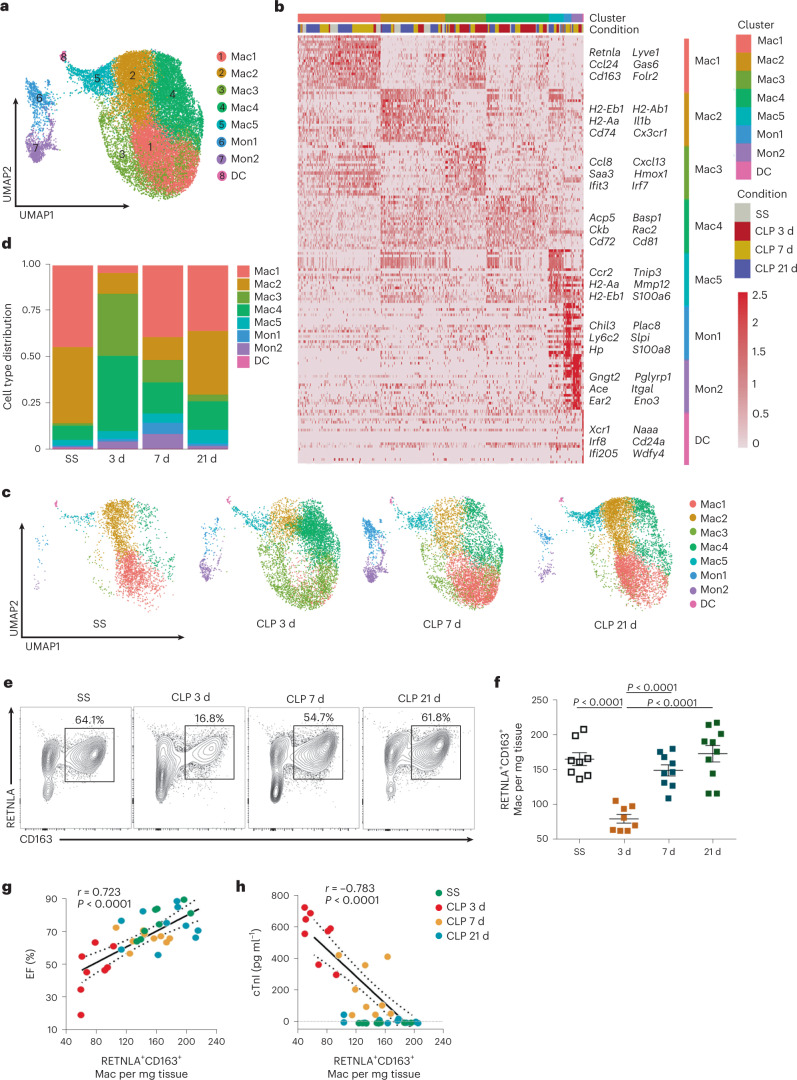
A distinct cardiac macrophage subset is associated with the recovery of SICM. **a**, UMAP plots of 24,058 monocyte-macrophages allocated to the eight clusters from Fig. 1c. **b**, Heat map showing top 20 cluster marker genes (top, color-coded by cluster and condition) with exemplar genes and cluster annotations labeled. **c**, UMAP plots of the monocyte-macrophage compartment from WT mice at SS, 3, 7 and 21 d after CLP, annotated by cluster as in **a**. **d**, Bar plots showing cluster distribution within the monocyte-macrophage compartment from WT mice after CLP. **e**, Representative contour plots showing flow cytometric analysis of cardiac CD45^+^CD11b^+^F4/80^+^CD163^+^RETNLA^+^ macrophages from WT mice after CLP. **f**, Quantification of CD163^+^RETNLA^+^ macrophages per mg of cardiac tissue. Bars show mean ± s.e.m. Two-sided *P* values were determined by one-way analysis of variance (ANOVA) with Tukey’s multiple comparison test. **g**,**h**, Correlation of cardiac CD163^+^RETNLA^+^ macrophage numbers with EF and cTnI levels. Data are presented as two-tailed Spearman’s rank correlation. Dashed lines represent 95% confidence intervals. Each symbol represents one animal (SS, *n* = 8 mice; CLP 3 d, *n* = 8 mice; CLP 7 d, *n* = 9 mice; CLP 21 d, *n* = 10 mice) (**f**,**g**). Results represent four independent experiments in this figure (Extended Data Fig. 3). Source data

**Extended Data Fig. 3:**
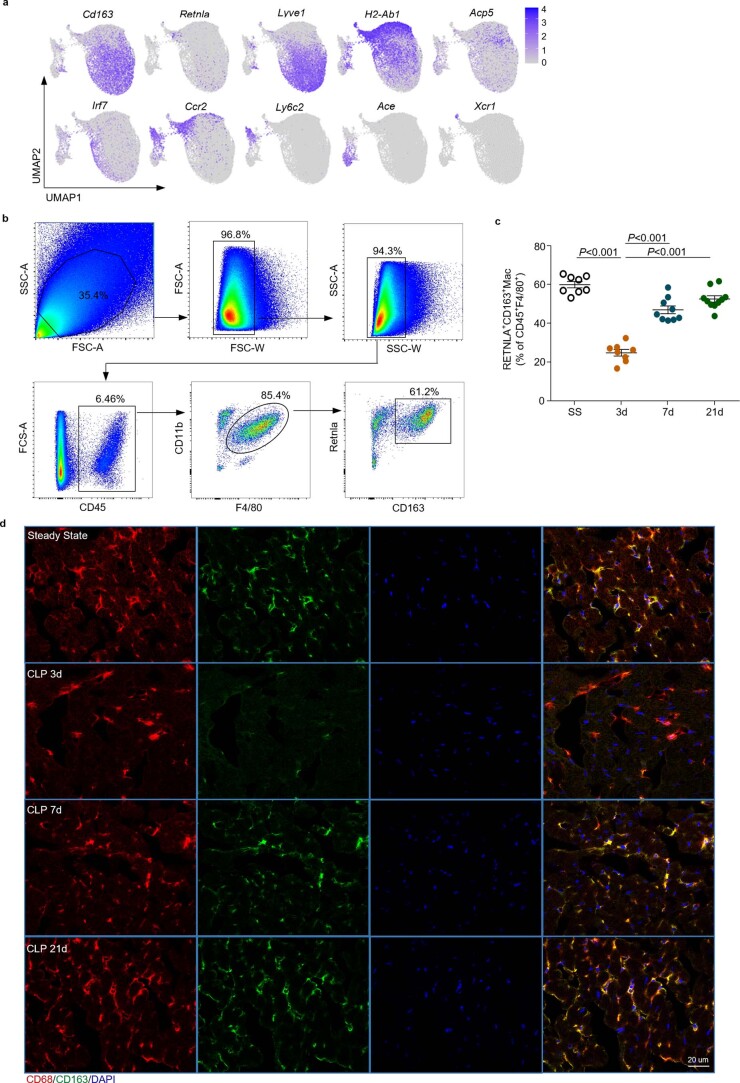
Identification of Mac1 subset in the septic heart, related to Fig. 2. a, UMAP plots showing the expression of selected marker genes for the monocyte-macrophage subpopulations. b, Gating strategy to identified Mac1 cells (CD45^+^CD11b^+^F4/80^+^CD163^+^RETNLA^+^). c, Graph showing percentages of CD163^+^RETNLA^+^ cardiac macrophages during sepsis progression (SS, n = 8 mice; CLP 3 d, n = 8 mice; CLP 7 d, n = 9 mice; CLP 21 d, n = 10 mice). Each symbol represents one animal. Bars show mean ± SEM. Two-sided P values were determined by one-way ANOVA with Games-Howell’s multiple comparisons test. d, Representative immunofluorescence images showing the presence of CD163 (green), CD68 (red), and nuclei (blue) in cardiac tissues during sepsis progression. n = 6 mice per group. Scale bars, 20 μm. Exact P values are shown. Results represent four independent experiments (c,d). SS, steady state. Source data

Given that the monocyte-macrophage compartment represents the largest and most significantly altered population of cardiac immune cells during sepsis, we performed an unsupervised cluster analysis to investigate the heterogeneity and role of cardiac monocyte-macrophages. Five macrophage subclusters (Mac1–5), two monocyte subclusters (Mon1 and Mon2) and a dendritic cell (DC) subcluster were identified (Fig. 2a). Mac1 was characterized by the expression of *Retnla*, *Lyve1*, *Cd163* and *Folr2*, which resembled the signatures of cardiac tissue-resident macrophages^19^. Mac2 expressed relatively high levels of antigen-presentation genes, the characteristics of which were consistent with the previously reported major histocompatibility complex (MHC)-II cluster^19,20^. Mac3 was enriched in *Cxcl13*, *Ccl8*, *Saa3* and a few IFN-stimulated genes (*Ifit3* and *Irf7*). This cluster might be pro-inflammatory and contained IFN-inducible cells^21^. Mac4 was identified by the expression of *Cd72* and *Acp5*, which were confirmed as markers of pathologically pro-inflammatory cells^22^. Mac5 corresponded to CCR2^+^ macrophages^12,19,23^, which also expressed high levels of antigen-presentation genes (*H2-Aa* and *H2-Eb1*). Mon1 was the classical monocyte cluster with high expression of *Ly6c2*, whereas Mon2 was the nonclassical monocyte cluster with low expression of *Ly6c2* (refs. 24, 25). The DC cluster was characterized by high levels of *Xcr1*, *Naaa* and *Irf8*, which were reported as markers of classical DCs^26^ (Fig. 2b and Extended Data Fig. 3a). We next compared the dynamic differences in relative proportions of the eight subclusters among different stages of SICM. Notably, the Mac1 and Mac2 subclusters were predominantly presented in hearts at steady state, whereas the levels of the Mac1 subset were dramatically reduced 3 d after CLP. The levels of Mac3 and Mac4, meanwhile, remarkably increased 3 d after CLP; however, as cardiac function gradually recovered 7 and 21 d after CLP, the Mac1 subcluster was restored (Fig. 2c,d). We also used flow cytometry and immunofluorescence to confirm scRNA-seq results by selecting CD163 and RETNLA as markers for Mac1. Consistently, the relative frequency and absolute numbers of Mac1 decreased 3 d after CLP and were restored 7 and 21 d after CLP (Fig. 2e,f and Extended Data Fig. 3b–d). Subsequently, correlation analysis found that the absolute numbers of Mac1 were positively correlated with EF of the heart and negatively correlated with serum cardiac troponin I (cTnI) levels during sepsis progression (Fig. 2g,h). Taken together, these data reveal a distinct CD45^+^CD11b^+^F4/80^+^CD163^+^RETNLA^+^ Mac1 subcluster that is associated with the restoration of cardiac dysfunction after septic stress.

### Developmental trajectory and lineage tracing of Mac1 subset

**Fig. 3:**
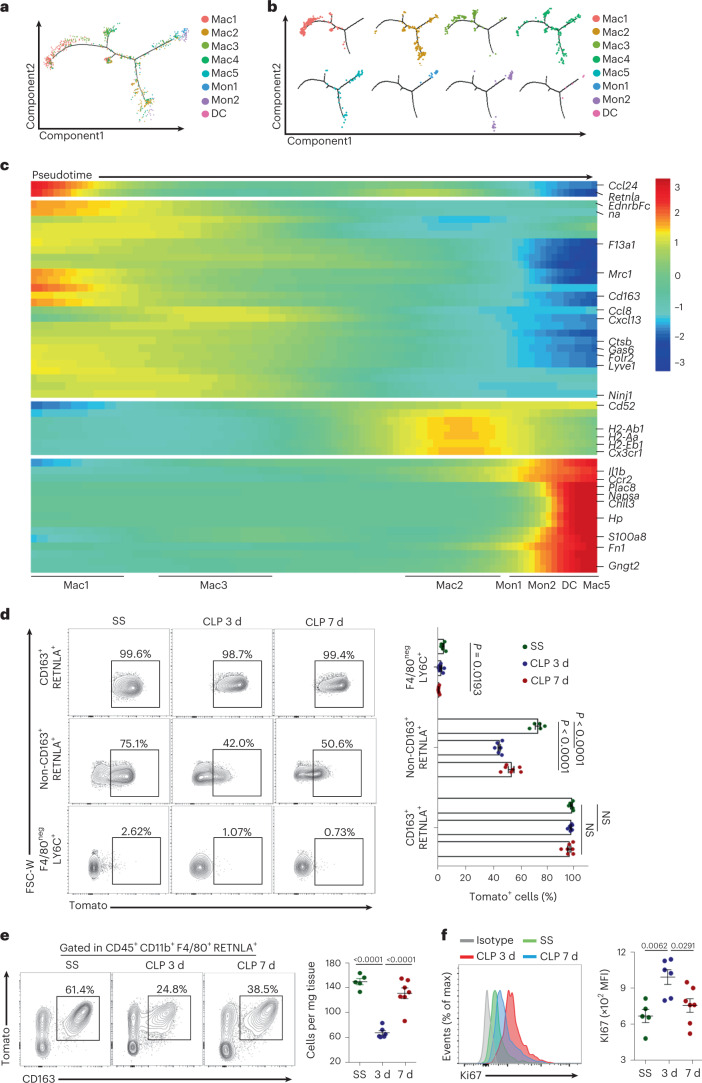
Developmental trajectory and lineage tracing track the fate of Mac1 subset in SICM. **a**, The Monocle prediction of the monocyte-macrophage developmental trajectory with Seurat’s cluster information in Fig. 2a mapped alongside. **b**, The Monocle prediction of monocyte-macrophage developmental trajectory with each Seurat-based cluster shown separately. **c**, Heat map of top 50 DEGs along with the pseudotime. The relative position of individual subsets across pseudotime is illustrated below. **d**, Representative contour plots showing the expression of tdTomato (CX3CR1) on gated CD45^+^CD11b^+^F4/80^+^CD163^+^RETNLA^+^ macrophages, CD45^+^CD11b^+^F4/80^+^ (non-CD163^+^RETNLA^+^) macrophages and CD45^+^CD11b^+^F4/80^−^LY6C^+^ monocytes. Graph showing the percentage of tdTomato^+^ cells isolated from hearts of WT mice at each time point along sepsis. NS, not significant. **e**, Representative contour plots showing the expression of tdTomato (CX3CR1) and CD163 on gated CD45^+^CD11b^+^F4/80^+^RETNLA^+^ macrophages and graph showing absolute numbers of CD45^+^CD11b^+^F4/80^+^CD163^+^RETNLA^+^tdTomato^+^ macrophages isolated from hearts of WT mice at each time point following sepsis. **f**, Representative histograms showing the expression of Ki67 in CD45^+^CD11b^+^F4/80^+^CD163^+^RETNLA^+^ macrophages and graph showing quantification of mean fluorescence intensity (MFI) of Ki67 at SS, 3 and 7 d after CLP. Dots represent individual subjects (**d**–**f**); SS, *n* = 5 mice; CLP 3 d, *n* = 6 mice; CLP 7 d, *n* = 7 mice. Data are shown as mean ± s.e.m.; two-sided *P* values were determined by one-way ANOVA, followed by Games-Howell’s (**d**) or Sidak’s (**d**–**f**) multiple comparison test; results represent three independent experiments (Extended Data Fig. 4). Source data

**Extended Data Fig. 4:**
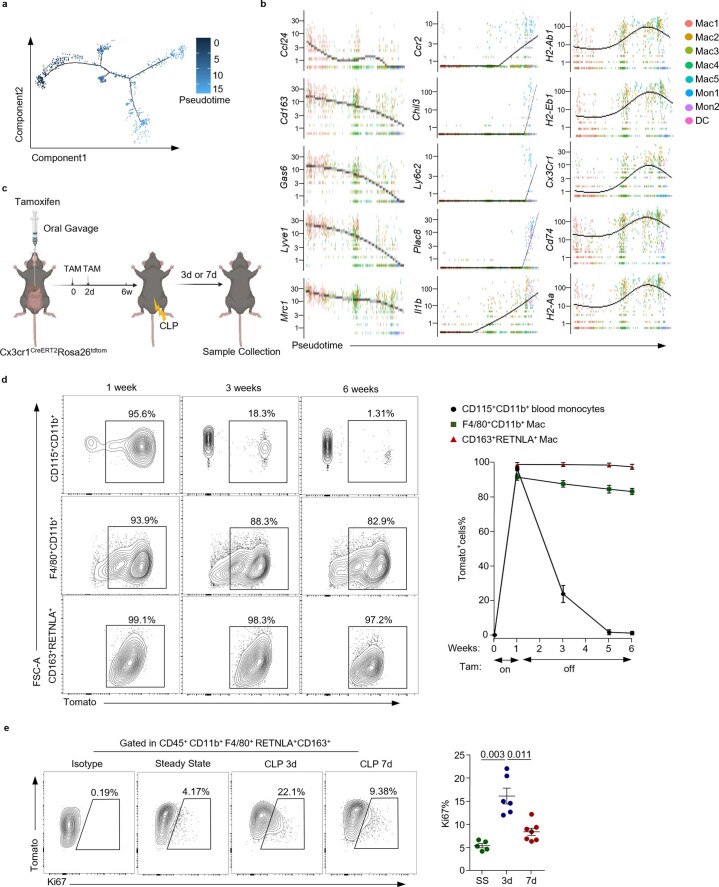
Developmental trajectory and lineage tracing track the fate of Mac1 subset in SICM, related to Fig. 3. a, Monocle prediction of monocyte-macrophage compartment developmental trajectory with pseudotime mapped along. b, Cluster-defining gene expression plotted along the pseudotime, with Seurat’s cluster mapped along. c, Schematic illustration of lineage tracking Mac1 cells in Cx3cr1^CreERT2^:Rosa26^tdTom^ mice. d, Representative contour plots showing the expression of tdTomato (CX3CR1) on gated CD115^+^CD11b^+^ blood monocytes, CD45^+^CD11b^+^F4/80^+^ cardiac macrophages, CD45^+^CD11b^+^F4/80^+^CD163^+^RETNLA^+^ macrophages and graph showing percentages of tdTomato-labeled cells in those cells (0 week, n = 4 mice; 1 week, n = 5 mice; 3 weeks, n = 5 mice; 5 weeks, n = 2 mice; 6 weeks, n = 3 mice). Bars are mean ± SEM. e, Representative contour plots showing the expression of Ki67 on gated CD45^+^CD11b^+^F4/80^+^CD163^+^RETNLA^+^ macrophages and graph showing percentages of Ki67 expressing in CD45^+^CD11b^+^F4/80^+^CD163^+^RETNLA^+^ macrophages at steady state (SS), 3 days and 7 days after CLP. Each symbol represents one animal. Data are presented as mean ± SEM. Two-sided P values were determined by one-way ANOVA with Games-Howell’s multiple comparisons test. Exact P values are shown. Results represent five (d) and two (e) independent experiments. Source data

To determine the potential relationship between monocytes and the Mac1 subcluster, we examined the dynamic immune states and cell transitions of the monocyte-macrophage compartment by the Monocle algorithm^27^. In pseudotime analysis, we downsampled monocyte-macrophages to 1,500 cells to provide a clear cell developmental trajectory. We observed that Mac1 cells localized at the beginning of the pseudotime trajectory, whereas Mon1, Mon2 and DC cells were at the end of the trajectory. In addition, the majority of Mac2 cells were at the terminus of the pseudotime trajectory, whereas Mac3 cells, similarly to the Mac1 cells, were localized at the initiating branch (Fig. 3a,b and Extended Data Fig. 4a). Tracking the alterations of gene expression across monocyte-macrophage compartments revealed developmental patterns defining the phenotype and function of macrophage subsets. In short, the Mac1 cluster was characterized by upregulated expression levels of *Cd163*, *Lyve1*, *Retnla*, *Mrc1*, *Gas6* and *Folr2* and by downregulated expression levels of monocyte genes (*Plac8* and *Chil3*). The Mon1, Mon2, Mac5 and DC clusters were characterized by upregulated expression of *Ccr2*, *Il1b*, *Chil3* and *Plac*8, whereas the Mac2 cluster was characterized by high expression of antigen-presenting genes (*H2-Aa* and *H2-Ab1*). These cell clusters displayed the reduced expression of the Mac1 gene profile (Fig. 3c and Extended Data Fig. 4b).

To confirm the above pseudotime analysis results, we further used a tamoxifen-inducible *Cx3cr1*^CreERT2–IRES–YFP^ mouse line crossed with *Rosa26*-stop-tdTomato reporter mice (termed *Cx3cr1*^CreERT2^:*Rosa26*^tdTom^; Extended Data Fig. 4c) to track Mac1 cells in SICM. tdTomato fluorescence signals of these reporter mice reliably distinguished CRMs from recruited monocyte-derived macrophages^19^. Around 95% of circulating monocytes (CD115^+^CD11b^+^) and ~89% cardiac macrophages (F4/80^+^CD11b^+^) were tdTomato^+^ 1 week after the first administration of tamoxifen. Three weeks after the administration of tamoxifen, circulating monocytes were gradually replaced by tdTomato^−^ monocytes, whereas their contribution to cardiac macrophages was negligible. At the time point of 5 weeks, almost 85% macrophages and ~98% CD163^+^RETNLA^+^ Mac1 cells in the heart retained tdTomato^+^, which was then maintained at similar levels (Extended Data Fig. 4d). Six weeks after administration of tamoxifen, *Cx3cr1*^CreERT2^:*Rosa26*^tdTom^ mice had CLP surgery. We observed that more than 95% of the CD163^+^RETNLA^+^ Mac1 cells remained as tdTomato^+^ 3 and 7 d after CLP (Fig. 3d). Accordingly, the proportion and the absolute numbers of tdTomato^+^ Mac1 cells in the septic heart were remarkably reduced 3 d after CLP and partly recovered 7 d after CLP (Fig. 3e). To determine the proliferation of Mac1 cells in vivo, Ki67 expression was assessed. tdTomato^+^ Mac1 cells exhibited high expression of Ki67 3 d after CLP compared to those at steady state, indicating that Mac1 cells presented an actively proliferative state under septic stress (Fig. 3f and Extended Data Fig. 4e). These data suggest that CD163^+^RETNLA^+^ Mac1 cells are self-renewing CRMs, independent of replenishment from circulating monocytes and display proliferative bursts during sepsis.

### TREM2 is essential for Mac1 cells remodeling during SICM

**Fig. 4:**
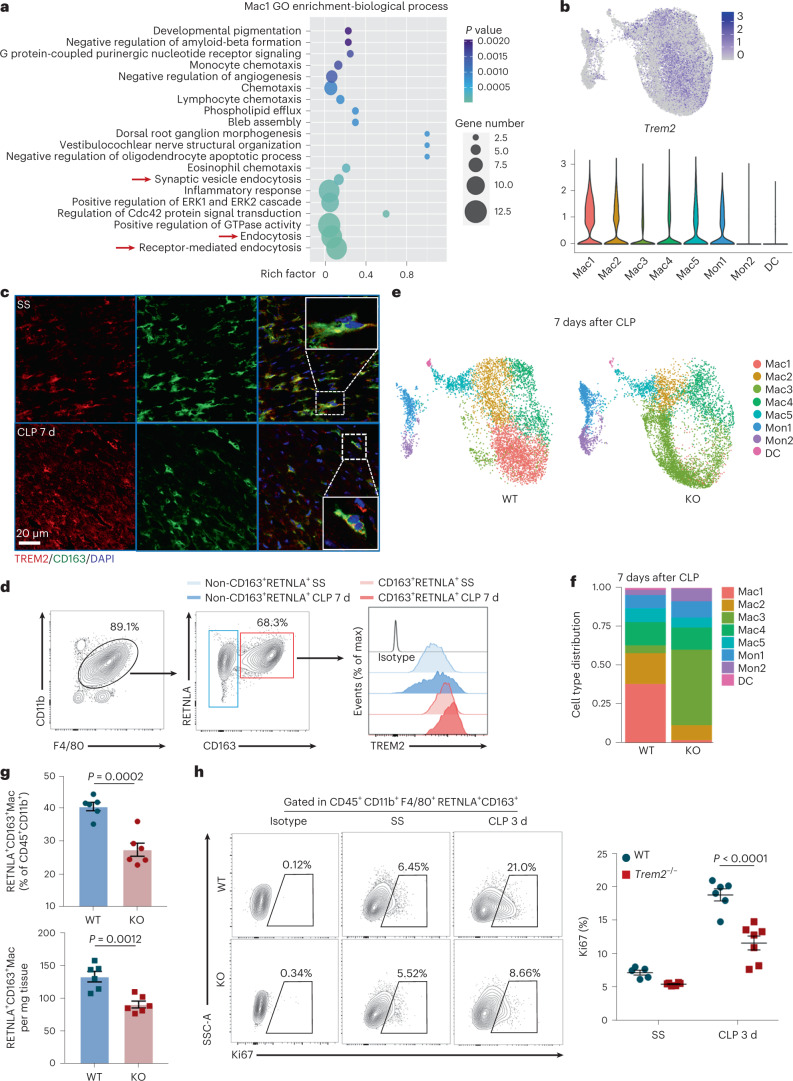
TREM2 is highly expressed on Mac1 subset and essential for Mac1 cells self-renewal in SICM. **a**, Dot plots showing the top 20 biological processes for the upregulated DEGs of Mac1 analyzed by GO. Red arrows indicate endocytosis-related pathways. **b**, UMAP plots and violin plots showing the expression of *Trem2* in the monocyte-macrophage compartment. **c**, Representative immunofluorescence images of CD163 (green), TREM2 (red) and nuclei (blue) in cardiac tissue from WT mice at SS (*n* = 5) and 7 d after CLP (*n* = 5). The dotted lines indicate CD163^+^TREM2^+^ macrophages. Scale bars, 20 μm. **d**, Representative histograms of flow cytometric analysis of TREM2 expression in cardiac CD45^+^CD11b^+^F4/80^+^CD163^+^RETNLA^+^ and CD45^+^CD11b^+^F4/80^+^(non-CD163^+^RETNLA^+^) macrophages from WT mice at SS and 7 d after CLP. **e**, UMAP plots showing cell clustering results corresponding to Fig. 2a for a total of 13,405 monocytes-macrophages in WT and *Trem2* knockout (KO) hearts at 7 d after CLP. **f**, Cluster distribution within the monocyte-macrophage subsets in WT and *Trem2*-KO hearts at 7 d after CLP. **g**, Percentages and absolute numbers of CD163^+^RETNLA^+^ macrophages analyzed by flow cytometry from hearts of WT (*n* = 6) and *Trem2*-KO (*n* = 6) mice at 7 d after CLP. **h**, Representative contour plots showing the expression of Ki67 on gated CD45^+^CD11b^+^F4/80^+^CD163^+^RETNLA^+^ macrophages and graph showing percentages of Ki67-expressing cells in WT (*n* = 5–6) and *Trem2*-KO (*n* = 6–7) hearts at SS and 3 d after CLP. Every symbol represents a mouse (**g**,**h**). Data are presented as mean ± s.e.m.; two-sided *P* values were determined by unpaired *t*-test (**g**) and one-way ANOVA with Sidak’s multiple comparisons test (**h**). Results represent three independent experiments (**c**,**d**,**g**,**h**). See also Extended Data Fig. 5. Source data

**Extended Data Fig. 5:**
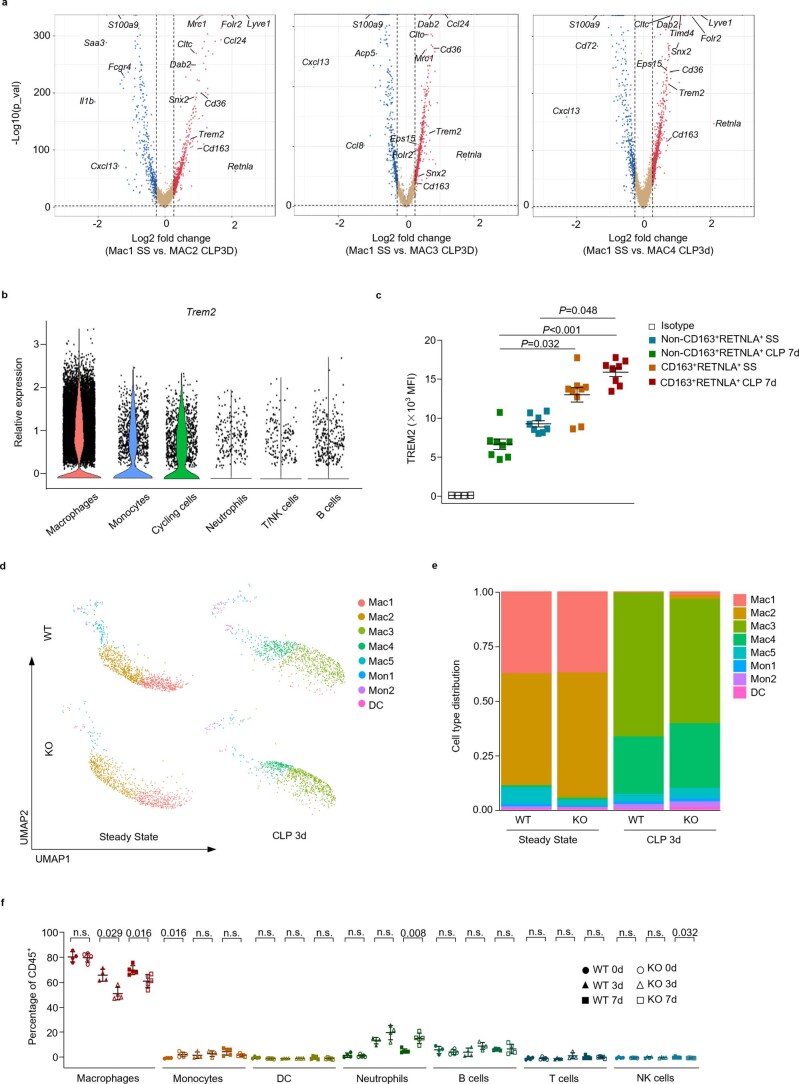
TREM2 is highly expressed on Mac1 subset and essential for Mac1 remodeling during SICM, related to Fig. 4. a, Volcano plots showing the gene expression fold change (x-axis, log2 scale) and p-value significance (y-axis, -log10 scale) in Mac1 subset of steady state (SS) versus Mac2 subset (left), Mac3 subset (middle) or Mac4 subset (right) of 3 days after CLP. Selected significant genes were labeled. Two-sided P values were determined by the Mann-Whitney U test. b, Violin plots showing the expression of *Trem2* in all defined immune cell types corresponding to Fig. 1c. c, Graph showing the quantification of mean fluorescence intensity (MFI) of TREM2 expression in CD45^+^CD11b^+^F4/80^+^CD163^+^RETNLA^+^ and CD45^+^CD11b^+^F4/80^+^(non-CD163^+^RETNLA^+^) cardiac macrophages at steady state and 7 days post-CLP (n = 8 mice for each group). Each symbol represents one animal. Data are presented as mean ± SEM. Two-sided P values were determined by Kruskal–Wallis with Dunn’s multiple comparison test. d, UMAP plots showing cell clustering results of the monocyte-macrophage compartment from hearts of WT littermate controls and *Trem2*-KO mice at steady state and 3 days after CLP. Monocyte-macrophages under each condition are downsampled to 1000 cells. The subpopulations obtained by Seurat were identical to those previously identified in Fig. 2a according to their marker genes. e, Bar plots showing cluster distribution of the monocyte-macrophage subsets in WT littermate controls and *Trem2*-KO mice at steady state and 3 days after CLP. f, Graph showing percentages of cardiac immune cells in WT controls and *Trem2*-KO mice at 0, 3 and 7 days post-CLP (n = 4-5 mice for each treatment). Every symbol represents a mouse. Data are presented as mean ± SEM, Two-sided P values were determined by Mann-Whitney U test, n.s., not significant. Results represent three independent experiments (c,f). Source data

We next sought to explore the functional characteristics of the Mac1 subset in SICM. Gene Ontology (GO) enrichment analysis revealed that 156 differentially expressed genes (DEGs) upregulated in Mac1 were related to phagocytosis and endocytosis in biological terms (Fig. 4a). Of note, phagocytosis-related gene *Trem2* was upregulated in Mac1 cells relative to other macrophages. (Extended Data Fig. 5a). We then examined the expression of *Trem2* in all cell clusters and observed that *Trem2* was particularly abundant in the Mac1 subset (Fig. 4b and Extended Data Fig. 5b). Immunofluorescence staining showed the expression of TREM2 on Mac1 cells as well as colocalization with CD163 (Fig. 4c). Flow cytometry analysis showed that CD163^+^RETNLA^+^ Mac1 cells expressed higher levels of TREM2 compared to other macrophages (Fig. 4d and Extended Data Fig. 5c). In addition, to determine the role of TREM2 for the remodeling of the macrophage subset in SICM, we performed CLP in *Trem2*-deficient (*Trem2*^−/−^) mice and wild-type (WT) littermate controls and analyzed their cardiac monocyte-macrophage compartments by scRNA-seq. Unsupervised cluster analysis of monocyte-macrophage compartments in *Trem2*^−/−^ and WT mice showed similar types and proportions of subclusters at steady state and 3 d after CLP, respectively (Extended Data Fig. 5d). Notably, we observed that the proportion of Mac1 cells both significantly decreased in WT and *Trem2*^−/−^ mice 3 d after CLP (Extended Data Fig. 5e). At day 7 after CLP, Mac1 cells recovered to normal counts in WT mice, but the proportion of Mac1 cells in *Trem2*^−/−^ mice could not be restored (Fig. 4e,f). Flow cytometry analysis confirmed that the percentage and absolute number of CD163^+^RETNLA^+^ Mac1 cells in *Trem2*^−/−^ mice did not recover at day 7 after CLP compared to WT controls (Fig. 4g). In contrast, the proportions of other cardiac immune cell compartments were not affected by *Trem2* deficiency, with the exception of reduction in the proportion of macrophages in *Trem2*^−/−^ mice 3 and 7 d after CLP and a modest increase in the proportion of neutrophils 7 d after CLP (Extended Data Fig. 5f). Moreover, we revealed that the proliferative capability of CD163^+^RETNLA^+^ CRMs was significantly decreased in *Trem2*^−/−^ mice compared to WT controls 3 d after CLP indicated by Ki67 expression (Fig. 4h). These results collectively suggest that TREM2 is a marker for the Mac1 subset and essential for the remodeling of Mac1 cells in septic hearts.

### Sepsis induces extracellular exopher accumulation in heart

**Fig. 5:**
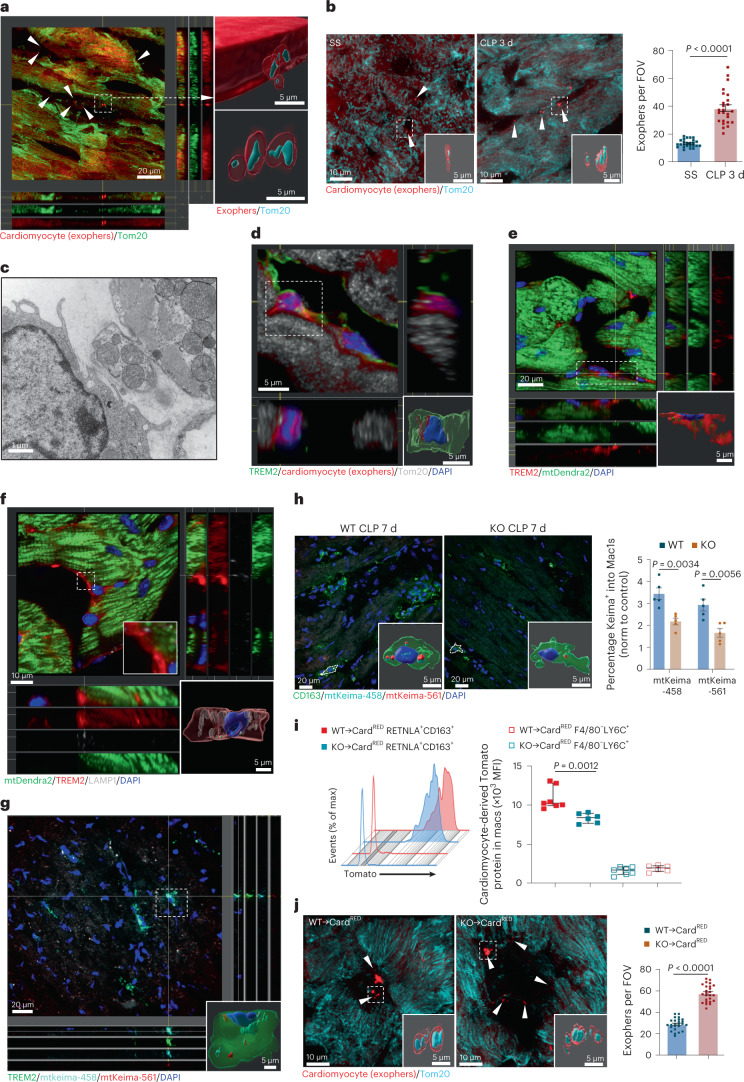
TREM2 promotes the uptake of cardiomyocyte-derived mitochondria by Mac1 in septic heart. **a**, Immunofluorescence images and 3D reconstruction showing presence of Tom20^+^ material in cardiomyocyte-derived exophers from hearts of septic Card^RED^ mice. **b**, Presence of Tom20^+^ material in cardiomyocyte-derived exophers from hearts of Card^RED^ mice at SS and 3 d after CLP. Graph showing exopher numbers per field of view (FOV); *n* = 6 per group. **c**, TEM image of a mononuclear cell taking up mitochondria by extended pseudopods in septic hearts (CLP 7 d). **d**, Images showing cardiomyocyte-derived exophers (red) containing mitochondria (Tom20, white) present in TREM2^+^ macrophages (green) from hearts of Card^RED^ mice (*n* = 4). **e**, Cardiomyocyte-derived mitochondria (mt-Dendra2, green) present in TREM2^+^ macrophages (red) from hearts of MitoCard mice (*n* = 4). **f**, Cardiomyocyte-derived mitochondria (mt-Dendra2, green) localized in lysosomes (LAMP1, white) of TREM2^+^ macrophages (red) in hearts of MitoCard mice (*n* = 3). **g**, TREM2^+^ macrophages (green) took up cardiomyocyte-derived mitochondria (mt-Keima-458, cyan) and some mitochondria in an acidic environment (mt-Keima-561, red) from hearts of AAV9-Tnnt2-mt-Keima infected mice. **h**, CD163^+^ macrophages (green) engulfed cardiomyocyte-derived mitochondria in hearts of WT and *Trem2*^−*/*−^ mice infected with AAV9-Tnnt2-mt-Keima, respectively. Graph showing percentages of mt-Keima^+^ mitochondria in CD163^+^ macrophages (*n* = 5 per group). For each animal, we randomly selected five visualization areas and five Mac1 cells were analyzed. The ratio of the area of mt-Keima mitochondria in a single Mac1 cell to the area of the same Mac1 cell was calculated. The ratio in the KO group was normalized to the ratio of the control group. **i**, The incorporation of cardiomyocyte-derived tdTomato protein in CD45^+^CD11b^+^F4/80^+^CD163^+^RETNLA^+^ macrophages and CD45^+^CD11b^+^F4/80^−^LY6C^+^ monocytes from WT → Card^RED^ or KO → Card^RED^ chimeras 7 d after CLP. Graph showing quantification of MFI of tdTomato (*n* = 6–7 per group). **j**, Presence of Tom20^+^ material (cyan) in cardiomyocyte-derived exophers (red) from hearts of WT → Card^RED^ and KO → Card^RED^ chimeras 7 d after CLP. Graph showing the exopher numbers per FOV (*n* = 6 per group). Scale bars are indicated in the images. Bars show as mean ± s.e.m. (**b**,**h**,**j**) and median with interquartile range (**i**). Two-sided *P* values were determined by unpaired *t-*test (**b**,**h**,**j**) and Mann–Whitney *U*-test (**i**). Results represent four (**b**), three (**d**–**f**) and two (**g**–**j**) independent experiments (Extended Data Figs. 6 and 7). Source data

**Extended Data Fig. 6:**
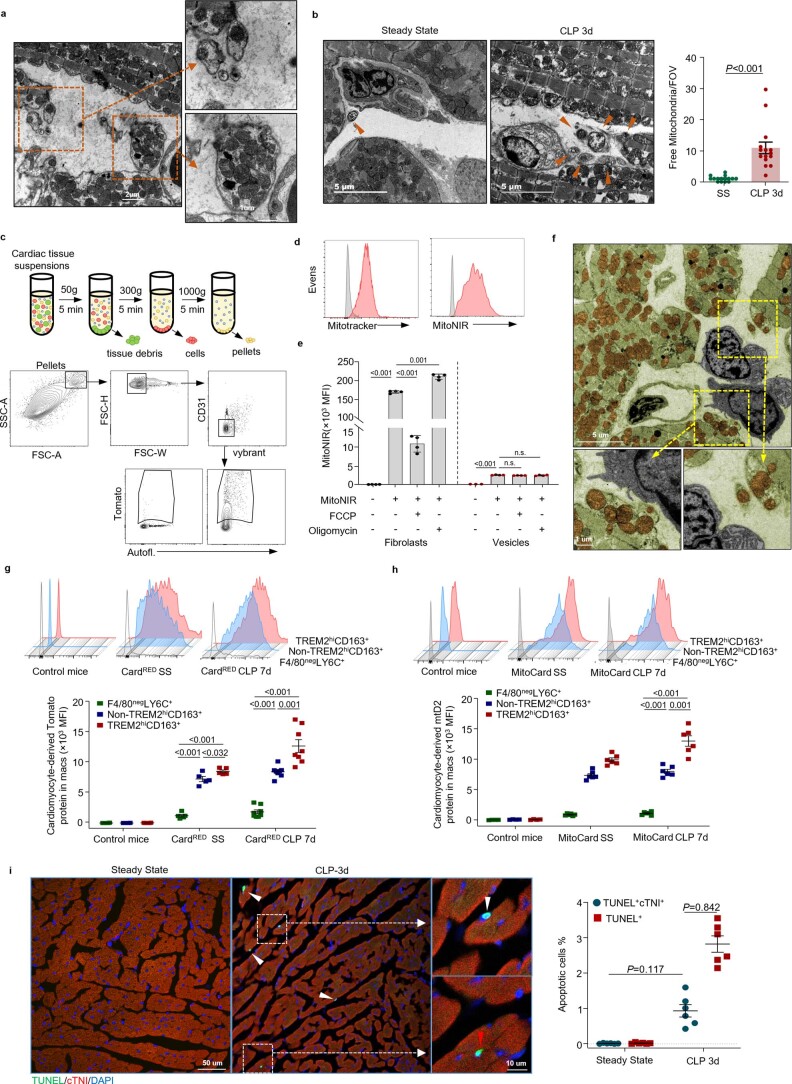
Sepsis induces extracellular damaged mitochondria accumulation and TREM2^hi^ Mac1 cells eliminate cardiomyocyte-derived mitochondria during sepsis, related to Fig. 5. a, TEM images showing vesicles in hearts of WT mice 3 days post-CLP. b, TEM images showing cardiac interstitial mitochondria vesicles (red arrowheads) of WT mice at steady state (SS) or 3 days post-CLP. Bar graph showing free mitochondria numbers per field of view (FOV). Each symbol represents a FOV. Five fields of view are randomly selected for assay from each animal (n = 3). c, Schematic illustration of the gating strategy for collecting vesicles from hearts of mice. d, Flow cytometric analysis of purified vesicles stained with the mitochondria-selective probes MitoTracker Green and MitoNIR (n = 3 per group). e, Mitochondrial membrane potential in cardiac vesicles and cultured fibroblasts as assessed by MFI levels of MitoNIR in basal conditions and in the presence of depolarizing (FCCP) or hyperpolarizing (Oligomycin) agents (n = 3-4 per group). f, Pseudo-colored representative TEM images of two mononuclear cells (grey) taking up vesicles containing mitochondria (brown) from cardiomyocytes (green) of WT mice 7 days post-CLP. g, The incorporation of cardiomyocyte-derived tdTomato protein in CD45^+^CD11b^+^F4/80^+^CD163^+^RETNLA^+^ macrophages, CD45^+^CD11b^+^F4/80^+^ (non-CD163^+^RETNLA^+^) macrophages and CD45^+^CD11b^+^F4/80^-^LY6C^+^ monocytes from αMHC^Cre/+^ (control) and αMHC^Cre/+^:Rosa26^TdTom^ (Card^RED^) mice at SS and 7 days post-CLP (control mice, n = 4; Card^RED^ SS, n = 5; Card^RED^ CLP 7d, n = 8). h, The incorporation of cardiomyocyte-derived mt-Dendra2 protein in CD45^+^CD11b^+^F4/80^+^CD163^+^RETNLA^+^ macrophages, CD45^+^CD11b^+^F4/80^+^(non-CD163^+^RETNLA^+^) macrophages and CD45^+^CD11b^+^F4/80^-^LY6C^+^ monocytes from αMHC^Cre/+^ (control) and αMHC^Cre/+^:mtD2^Flox/Flox^ (MitoCard) mice at SS and 7 days post-CLP (control mice, n = 4; MitoCard SS, n = 6; MitoCard CLP 7d, n = 6). i, Proportions of TUNEL^+^ and TUNEL^+^cTnI^+^ cells in hearts of WT mice at SS and 3 days post-CLP (n = 6 mice per group). All scale bars are indicated in the images. Data are presented as mean ± SEM (b,e,g,h,i). Two-sided P values were determined by Mann-Whitney U test (b), or by Kruskal–Wallis with Dunn’s multiple comparison test (e,g,h,i). Results represent three (a,b,d,g-i) and two (e,f) independent experiments. Source data

Cardiomyocytes ejected dysfunctional mitochondria in dedicated vesicles referred to as ‘exophers’, which are characterized as 3.5 ± 0.1 μm in mean diameter and 31.0 ± 2.5 μm^3^ in mean volume^14^, similar to structures described in *Caenorhabditis elegans* neurons^28^. Cardiac macrophages are indispensable in maintaining heart homeostasis by cleaning these subcellular particles and defective mitochondria^14^. Our data suggested that homeostasis of macrophages in septic hearts was disrupted. We speculated that disrupted homeostasis of macrophages could impair the removal of cardiomyocyte-derived damaged mitochondria during sepsis. First, *αMHC*^Cre^ mice were crossed with *Rosa26*^TdTom^ mice and only the cardiomyocytes of generated mice (referred as Card^RED^ mice) expressed red fluorescence. We observed that the accumulation of some subcellular particles, defined as exophers, colocalized with mitochondrial protein Tom20 in the extracellular space of septic Card^RED^ mice hearts (Fig. 5a,b and Supplementary Video 1). Transmission electron microscopy (TEM) analysis revealed a significant accumulation of free mitochondria in the periphery of cardiomyocytes 3 d after CLP (Extended Data Fig. 6a,b). In addition, these exophers were purified with flow cytometry^14^. We observed that cardiac exophers were positive for mitochondrial probes MitoTracker Green and MitoNIR (Extended Data Fig. 6c,d), also displayed the reduced membrane potential and lost responsiveness to hyperpolarizing agents (Extended Data Fig. 6e). These results suggest that sepsis causes the accumulation of dysfunctional mitochondria ejected by cardiomyocytes in the extracellular space of the heart.

### TREM2^hi^ Mac1 cells eliminate cardiac exophers during sepsis

Given that free mitochondria and mitochondrial DNA (mtDNA) can elicit cardiac damage, elimination of exophers containing cardiomyocyte-derived mitochondria is important to maintain cardiac homeostasis and restore optimal cardiac function^29,30^. We next explored the mechanism for the clearance of dysfunctional mitochondria in the septic heart. TEM images showed that some cells adjacent to cardiomyocytes displayed a similar size and morphology to macrophages. These macrophages stretched out pseudopods, which surrounded the mitochondria-containing vesicles in the periphery of septic cardiomyocytes (Fig. 5c and Extended Data Fig. 6f). Consistently, confocal and three-dimensional (3D) images showed that TREM2^+^ cell-engulfed exophers (red) contained mitochondria in septic hearts of Card^RED^ mouse (Fig. 5d and Supplementary Video 2).

To quantify the phagocytic activity of TREM2^hi^ Mac1 (CD45^+^CD11b^+^F4/80^+^CD163^+^) cells, flow cytometry analysis with the hearts of Card^RED^ mice revealed the higher incorporation of cardiomyocyte-derived tdTomato protein in Mac1 cells compared to non-Mac1 cells (Extended Data Fig. 6g). Further, cardiomyocyte-specific mitochondria reporter mice (MitoCard) were generated by crossing mtD2^Flox/Flox^ with *αMHC*^Cre/+^ mice^31^ and confocal images showed that cardiomyocyte-derived mitochondria localized in TREM2^+^ macrophages (Fig. 5e and Supplementary Video 3). Flow cytometry analysis also showed that TREM2^hi^ Mac1 cells incorporated more cardiomyocyte-derived mitochondria during SICM (Extended Data Fig. 6h). In addition, we performed TUNEL staining with hearts of septic WT mice and found that the percentage of TUNEL^+^cTnI^+^ cells in total cells (TUNEL^+^cTnI^+^/4,6-diamidino-2-phenylindole (DAPI)) was very low (around 1%) on day 3 after sepsis (Extended Data Fig. 6i). These results excluded the possibility that TREM2^hi^ cells incorporate apoptotic material derived from the septic heart.

**Extended Data Fig. 7:**
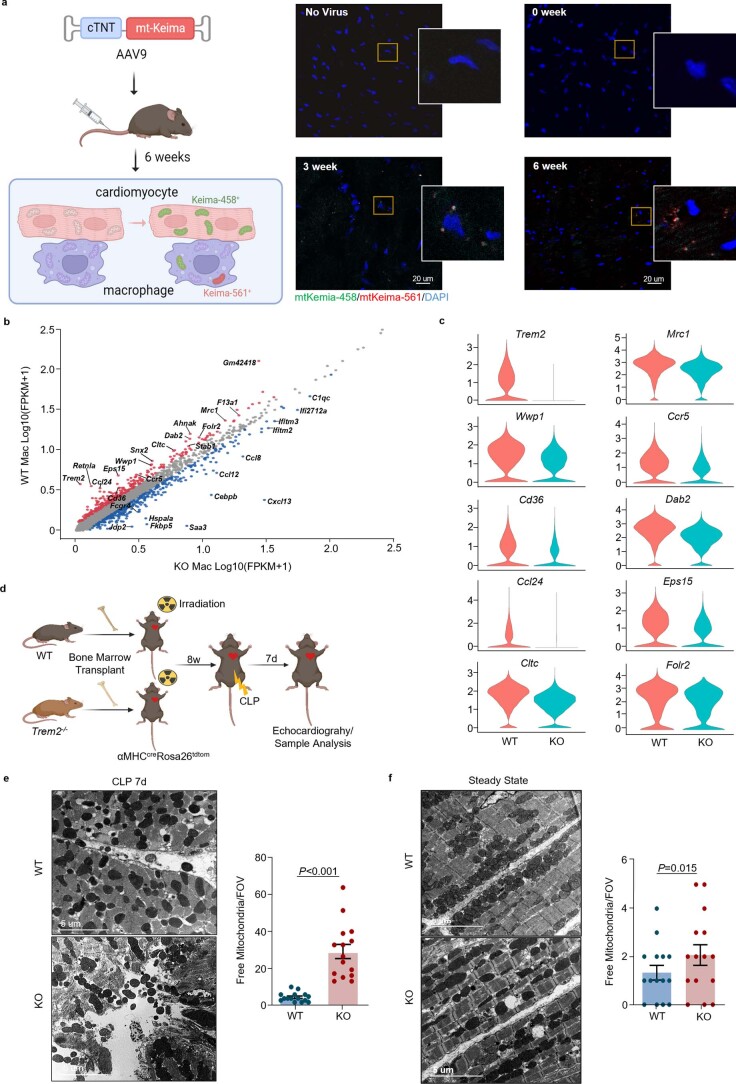
*Trem2* deficiency impairs the uptake of cardiomyocyte-derived mitochondria by Mac1 subset in septic heart, related to Fig. 5. a, Schematic illustration of the cardiomyocyte mitochondria labeled with AAV9-Tnnt2-mt-Keima virus. Right panel showing the mt-Keima fluorescence signals in hearts of WT mice at weeks 0, 3 and 6 after virus infection respectively. Keima-tagged mitochondria were indicated by different fluorescence in neutral (Keima 458 nm; green) and acidic (Keima 561 nm; red) environments. Scale bars, 20 μm. b, Scatter plots showing the average expression levels (log10(FPKM+1) scale) of total macrophage populations from WT littermate controls (y-axis) and *Trem2*^-/-^ mice (x-axis). c, Violin plots showing the selected endocytic gene expression in total macrophage from WT littermate controls and *Trem2*^-/-^ mice. d, Study protocol of bone marrow transplantation from WT or *Trem2*^-/-^ donors into Card^RED^ recipient mice. e,f, Representative TEM images showing cardiac interstitial mitochondria vesicles from WT and *Trem2*^-/-^ mice at steady state (f) and 7 days post-CLP (e), Scale bars, 5 μm. Bar graph showing the quantification of free mitochondria numbers per field of view (FOV). Each symbol represents a FOV (n = 3 mice for each treatment). Five fields of view are randomly selected for assay from each animal. Data are presented as mean ± SEM (e,f). Two-sided P values were determined by Mann-Whitney U test (e,f). Results represent two independent experiments (e,f). Source data

To elucidate the fate of cardiomyocyte-derived mitochondria in Mac1 cells, we performed immunostaining of TREM2 with lysosome marker Lamp1 with the heart sections of septic MitoCard mice and observed that mt-Dendra2 signals in TREM2^+^ cells partially localized in LAMP1^+^ lysosomes (Fig. 5f and Supplementary Video 4). Next, we packaged adeno-associated virus serotype 9 (AAV9)-Tnnt2-mt-Keima virus and intramyocardial injection of AAV9 virus was performed to ensure the specific expression of Keima protein in the mitochondria (mt-Keima) of cardiomyocytes (Extended Data Fig. 7a)^32^. Immunofluorescence staining revealed that abundant mt-Keima^+^ particles concentrated in TREM2^+^ macrophages (Fig. 5g). Keima is pH-sensitive and its peak of the excitation spectrum can shift from the shorter (green, in the neutral environment) to longer (red, in the acidic environment) wavelength^33^. Video and 3D images showed that mt-Keima in TREM2^+^ macrophages exhibited both green and red fluorescence signals in the hearts of septic WT mice injected with AAV9-Tnnt2-mt-Keima virus (Fig. 5g and Supplementary Video 5), suggesting that mitochondria taken up by Mac1 cells may be delivered to the acidic lysosomal environment. Together, these data indicate that TREM2^hi^ Mac1 cells have robust activity in eliminating of cardiomyocyte-derived dysfunctional mitochondria.

### *Trem2* deficiency impairs defective mitochondria removal

scRNA-seq data analysis revealed that the majority of the endocytic gene signatures such as *Wwp1*, *Ccr5*, *Cd36*, *Eps15*, *Cltc*, *Mrc1*, *Ccl24*, *Dab2* and *Folr2* in the whole macrophage population decreased in *Trem2*^−*/*−^ mice compared to WT cells (Extended Data Fig. 7b,c). We then examined the effect of TREM2 on the uptake of cardiomyocyte-derived mitochondria by macrophages. The intramyocardial injection of AAV9-Tnnt2-mt-Keima virus was carried out with WT and *Trem2*^−*/*−^ mice, respectively. *Trem2*^−/−^ septic mice displayed the reduced uptake of cardiomyocytic mitochondria and Keima by CD163^+^ macrophages (Fig. 5h and Supplementary Video 5). Next, we transplanted bone marrow cells from WT or *Trem2*^−/−^ mice into irradiated Card^RED^ recipient mice and 8 weeks later, CLP surgery was performed (Extended Data Fig. 7d). We found that WT → Card^RED^ CD163^+^RETNLA^+^ Mac1 cells scavenged more cardiomyocyte-derived material than *Trem2*^−*/*−^→Card^RED^ CD163^+^RETNLA^+^ Mac1 cells 7 d after CLP (Fig. 5i). Consistently, immunofluorescence showed that more cardiomyocyte-derived mitochondria accumulated in the heart of *Trem2*^−*/*−^→Card^RED^ chimeras (Fig. 5j). In addition, *Trem2-*deficient mice exhibited severe accumulation of free mitochondria in the extracellular space of cardiomyocytes 7 d after CLP (Extended Data Fig. 7e), but this was not found in *Trem2*-deficient mice at steady state (Extended Data Fig. 7f). These data suggest that TREM2 facilitates the scavenging of damaged mitochondria by Mac1 cells in septic hearts.

### *Trem2* deficiency exacerbates cardiac dysfunction in sepsis

**Fig. 6:**
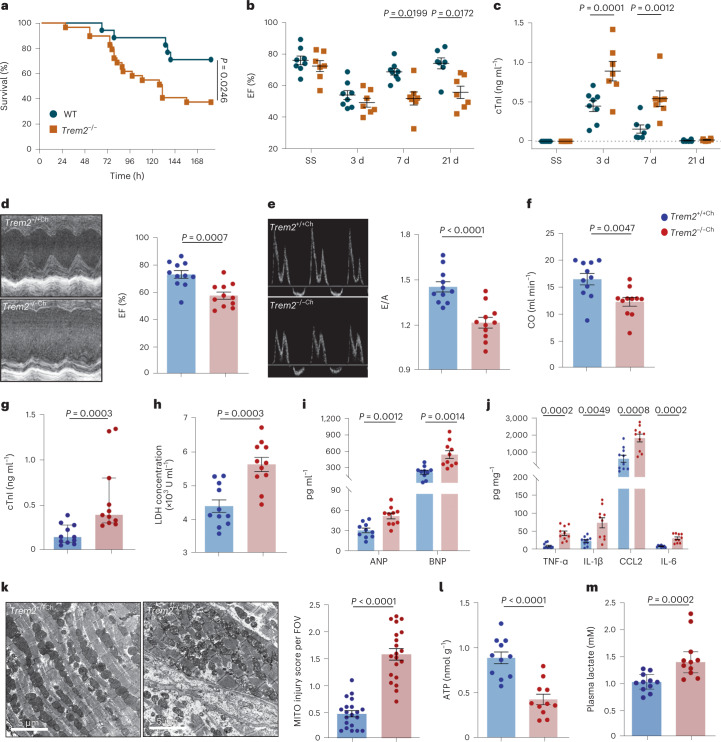
*Trem2* deficiency exacerbates the cardiac dysfunction following sepsis. **a**, Survival analysis of WT (*n* = 18) and *Trem2*^−/−^ (*n* = 30) mice after CLP performance was monitored for 7 d. **b**,**c**, WT and *Trem2*^−/−^ mice were subjected to CLP and cardiac function was examined at SS and 3, 7 and 21 d after CLP. Graph showing EF % (**b**) measured by echocardiography (*n* = 7–8 mice for each group) and the levels of cTnI (**c**) in the serum (*n* = 5–8 mice for each group). **d**, Representative M-mode echocardiography images and EF % of *WT* → *WT chimeras (Trem2*^+/+Ch^*) and Trem2*^−/−^*→WT chimeras (Trem2*^−/−Ch^*)* 7 d after CLP. **e**, Representative continuous-wave Doppler echocardiography images and E/A ratio of *Trem2*^+/+Ch^ and *Trem2*^−/−Ch^ chimeras 7 d after CLP. **f**, Graph showing CO measured by echocardiography. **g**,**h**, Graphs showing the levels of cTnI (**g**) and LDH (**h**) in the serum of *Trem2*^+/+Ch^ and *Trem2*^−/−Ch^ chimeras 7 d after CLP (**d**–**h**, *n* = 11 mice for each group). **i**, Graphs showing protein levels of ANP and BNP in the serum of *Trem2*^+/+Ch^ and *Trem2*^−/−Ch^ chimeras 7 d after CLP. **j**, Graphs showing protein levels of tumor necrosis factor (TNF)-α, IL-1β, IL-6 and CCL2 in the heart tissues of *Trem2*^+/+Ch^ and *Trem2*^−/−Ch^ chimeras 7 d after CLP (**i**,**j**, *n* = 10 mice for each group). **k**, Representative TEM images (left) and mitochondria injury score (right) of *Trem2*^+/+Ch^ and *Trem2*^−/−Ch^ chimeras 7 d after CLP. Scale bars, 5 μm. Bar graph showing mitochondria injury score per FOV. Each symbol represents a FOV of TEM images. Each group has four mice and five FOVs were randomly selected for assay from each animal. **l**,**m**, Graphs showing the levels of ATP (**l**) in heart tissue lysates and the levels of serum lactate (**m**) of *Trem2*^+/+Ch^ and *Trem2*^−/−Ch^ chimeras 7 da after CLP (**l**,**m**, *n* = 11 mice for each group). Each symbol represents one animal (**b**–**j**,**l**,**m**). Bars show as mean ± s.e.m. (**b**–**f**,**h**–**l**) and median with interquartile range (**g**,**m**). Two-sided *P* values were determined by Kaplan–Meier log-rank test (**a**), two-way ANOVA with Sidak’s multiple comparisons test (**b**,**c**), unpaired *t*-test (**d**–**f**,**h**–**k**) and Mann–Whitney *U*-test (**g**,**m**). Results represent at least four (**b**,**c**) and three (**d**–**m**) independent experiments (Extended Data Fig. 8). Source data

**Extended Data Fig. 8:**
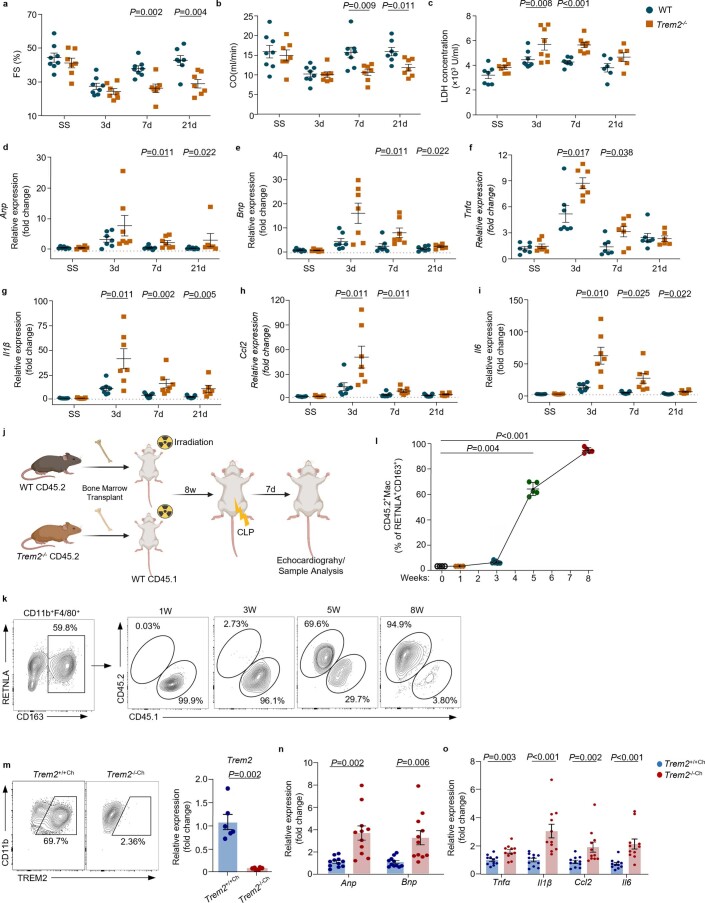
*Trem2* deficiency exacerbates cardiac dysfunction in sepsis, related to Fig. 6. a-i, WT and *Trem2*^-/-^ mice were subjected to CLP and cardiac function was examined at steady state (SS) and 3, 7 and 21 days post-CLP. Graph showing FS% (a); CO (b) measured by echocardiography; Graph showing the levels of LDH in the serum (c); Graphs showing mRNA levels of *Anp* (d), *Bnp* (e), *Tnfα* (f), *Il-1β* (g), *Ccl2* (h) and *Il-6* (i) in the heart tissue lysates (a-b, n = 7–8 mice; c, n = 6-8 mice; d-i, n = 6-7 mice for each treatment). j, Study protocol of bone marrow transplantation (BMT) from WT and *Trem2*^-/-^ donors (CD45.2 background) into WT recipients (CD45.1 background). k, Representative contour plots of CD45.1 and CD45.2 expressions on gated CD45^+^CD11b^+^F4/80^+^CD163^+^RETNLA^+^ Mac1 subset in recipient mice 1, 3, 5 and 8 weeks after BMT. l, Graph showing percentages of CD45.2^+^ cells in CD11b^+^F4/80^+^CD163^+^RETNLA^+^ Mac1 subset after BMT (n = 4-7 mice for each group).m, Representative contour plots of TREM2 expression on gated CD45^+^CD11b^+^F4/80^+^CD163^+^ macrophages in hearts of both WT→WT chimeras (*Trem2*^+/+C^^h^) and *Trem2*^-/-^→WT chimeras (*Trem2*^-/-Ch^) 8 weeks after BMT. Graph showing *Trem2* mRNA levels in heart tissue lysates (n = 6 mice for each group). n, Graphs showing mRNA levels of *Anp* and *Bnp* in the heart tissue lysates 7 days post-CLP (n = 11 mice for each group). o, Graphs showing mRNA levels of *Tnfα*, *Il1β*, *Ccl2* and *Il6* in the heart tissue lysates 7 days post-CLP (n = 11 mice for each group). Each symbol represents one animal. All data are presented as mean ± SEM. Two-sided P values were determined by Mann-Whitney U test (a-i,m-o) or by Kruskal-Wallis with Dunn’s multiple comparison test (l). Results represent at least four (a-i,l) and three (m-o) independent experiments. Source data

To determine the physiological importance of TREM2^hi^ Mac1 cells in SICM, we compared survival rate, cardiac systolic function, cardiac injury markers and inflammation between WT and *Trem2*^−/−^ mice after CLP. As shown in Fig. 6a, the mortality of *Trem2*^−*/*−^ septic mice was notably increased. Moreover, echocardiography assay revealed that *Trem2*^−*/*−^ mice had worse cardiac function indicated by lower EF, fractional shortening (FS) and cardiac output (CO), especially 7 and 21 d after CLP, compared to WT littermate controls (Fig. 6b and Extended Data Fig. 8a,b). The concentrations of cTnI and LDH in the serum as well as messenger RNA levels of *Anp* and *Bnp* in heart tissues were all increased in *Trem2*^−*/*−^ septic mice (Fig. 6c and Extended Data Fig. 8c–e). Also, *Trem2* deficiency significantly elevated mRNA levels of pro-inflammatory mediators after CLP (Extended Data Fig. 8f–i).

Bone-marrow-derived macrophages contribute to the resident microglia pool in chimeric mice^34,35^. Irradiated recipients (CD45.1 background) were transplanted with bone marrow cells from WT or *Trem2*^−*/*−^ mice (CD45.2 background), respectively (Extended Data Fig. 8j). Flow cytometry assay showed that the efficiency of Mac1 cells reconstitution was over 90% in irradiated chimeras 8 weeks after bone marrow transplantation (BMT) (Extended Data Fig. 8k, l). Flow cytometry and real-time–PCR results revealed that *Trem2*^−/−^→WT chimeras (*Trem2*^−/−Ch^) displayed lower TREM2 protein level in cardiac macrophages and *Trem2* mRNA level in heart tissues than WT → WT chimeras (*Trem2*^+/+Ch^), respectively (Extended Data Fig. 8m). Eight weeks after BMT, chimeric mice were subjected to CLP. We found that *Trem2*-specific deficiency in macrophages exacerbated cardiac dysfunction, myocardial injury and cardiac inflammation following sepsis (Fig. 6d–j and Extended Data Fig. 8n,o). In addition, *Trem2*^−/−Ch^ chimeras exhibited aggravated mitochondrial damage (Fig. 6k), reduced ATP levels (Fig. 6l) and increased lactate levels (Fig. 6m). These results suggest that TREM2 is essential for Mac1 to protect the cardiac function after sepsis.

### Transplantation of TREM2^hi^ Mac1 cells protects SICM

**Fig. 7:**
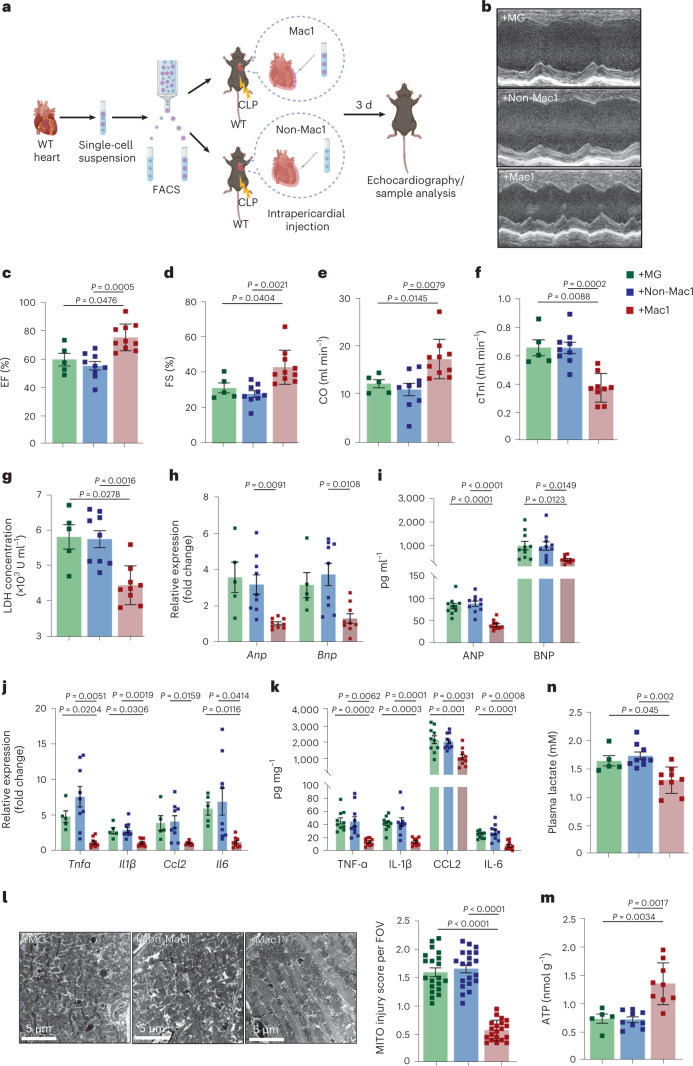
Intrapericardial administration of TREM2^hi^ Mac1 cells protects SICM. **a**, Schematic illustration of TREM2^hi^ Mac1 cells transplantation in WT mice. TREM2^hi^ Mac1 (CD45^+^CD11b^+^F4/80^+^CD163^+^RETNLA^+^) and non-Mac1 (CD45^+^CD11b^+^ F4/80^+^ and non-CD163^+^RETNLA^+^) cells isolated from WT mice were mixed with MG and transplanted into the pericardial cavity of WT mice (2 × 10^5^ cells per animal) immediately after CLP. Control mice were injected with MG only. Mice were killed and analyzed 3 d after the transplantation. **b**–**e**, Representative M-mode echocardiography images (**b**) and graphs showing EF % (**c**), FS % (**d**) and CO (**e**) measured by echocardiography (**b**–**e**, +MG, *n* = 5 mice; +non-Mac1, *n* = 9 mice; +Mac1, *n* = 10 mice). **f**,**g**, Graphs showing levels of cTnI and LDH in the serum. **h**, Graphs showing mRNA levels of *Anp* and *Bnp* in the heart tissues. **i**, Graphs showing protein levels of ANP and BNP in the serum. **j**, Graphs showing mRNA levels of *Tnfα*, *Il1β*, *Ccl2* and *Il6* in heart tissues. **k**, Graphs showing protein levels of TNF-α, IL-1β, IL-6 and CCL2 in heart tissues. **l**, Representative TEM images (left) and mitochondria injury score (right). Scale bars, 5 μm. Each symbol represents a FOV. Each group has four mice and five FOV were randomly selected for assay from each animal. **m**, Graph showing the levels of ATP in heart tissue lysates. **n**, Graph showing the levels of serum lactate. (**f**–**k**,**m**,**n**, +MG, *n* = 5 mice; +non-Mac1, *n* = 9 mice; +Mac1, *n* = 9 mice). Each symbol represents one animal in **c**–**k**, **n**, **m**. Bars show as mean ± s.e.m. Two-sided *P* values were determined by one-way ANOVA followed by Games-Howell’s (**c**–**h**,**j**,**n**,**m**) or Tukey’s (**i**,**k**) multiple comparisons test. Results represent four independent experiments (**b**–**n**). See also Extended Data Figs. 9 and 10. Source data

**Extended Data Fig. 9:**
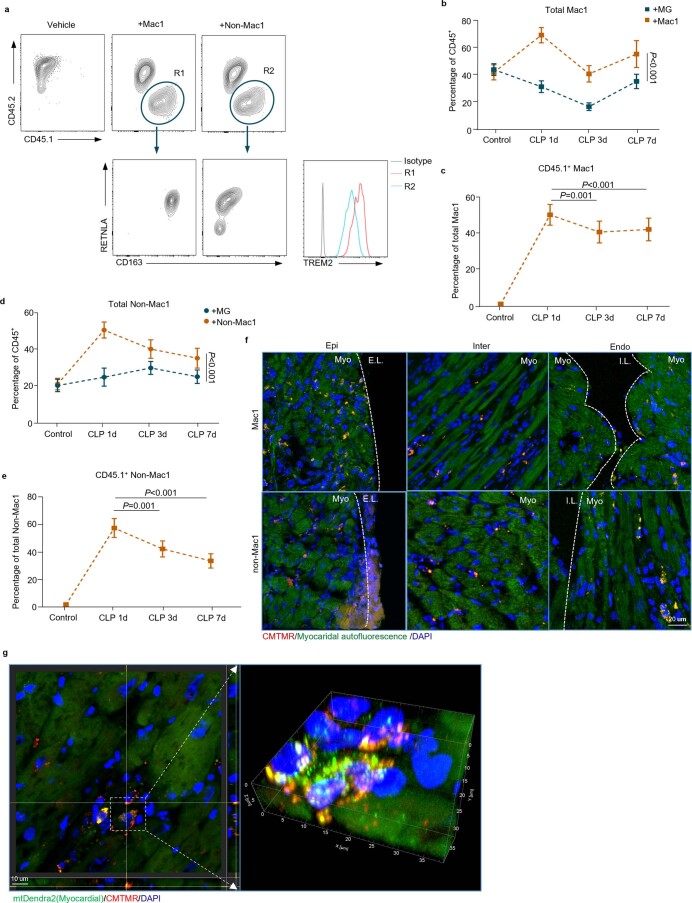
Transplanted cardiac macrophages are detected by flow cytometry and immunofluorescence, related to Fig. 7. a, TREM2^hi^ Mac1 cells (CD45^+^CD11b^+^F4/80^+^CD163^+^RETNLA^+^) and non-Mac1 (CD45^+^CD11b^+^F4/80^+^ and non-CD163^+^RETNLA^+^) cells were isolated from CD45.1 mice. Mac1 or non-Mac1 cells (2 × 10^5^ cells per mouse) were mixed with Matrigel (MG), and transplanted into the hearts of CD45.2 mice, respectively. Seven days after the transplantation, macrophages from hearts of recipients (CD45.2) were analyzed with flow cytometry assay. b,c, Graphs showing percentages of total Mac1 (b) and CD45.1^+^ Mac1 (c) cells within the CD45^+^ populations from hearts of recipients (CD45.2) at 1, 3, and 7 day(s) post the cell transplantation, respectively (n = 6 mice for each treatment). d,e, Graphs showing percentages of total non-Mac1 (d) and CD45.1^+^ non-Mac1 (e) cells within the CD45^+^ populations from hearts of recipients (CD45.2) at 1, 3, and 7 day(s) post the cell transplantation, respectively (n = 5-6 mice for each treatment). f,g, TREM2^hi^ Mac1 cells (CD45^+^CD11b^+^F4/80^+^CD163^+^RETNLA^+^) and non-Mac1 (CD45^+^CD11b^+^F4/80^+^ and non-CD163^+^RETNLA^+^) cells were obtained from the hearts of WT mice by FACS and stained by CMTMR. Then, both types of cells (2 × 10^5^ cells/mouse mixed with Matrigel) were respectively injected into the pericardial cavity of recipient mice immediately after CLP. f, Representative immunofluorescence images showing the transplanted cells in the epicardium (Epi), intermyocardium (Inter), and endocardium (Endo) of recipient mice at 3 days post the cell transplantation. CMTMR (red) signals indicating Mac1 or non-Mac1 cells; epicardial layer (E.L.); myocardium (Myo); inner layer (I.L.). Scale bar, 20 μm. g, Representative immunofluorescence images showing the transplanted CMTMR labeled Mac1 cells in MitoCard mice at 3 days post CLP. 3D reconstruction images of the dashed box area, illustrating that cardiomyocyte-derived mitochondria (mtDendra2^+^, green) presented in the transplanted cells (CMTMR^+^, red). Nuclei were shown in blue (DAPI). Scale bar, 10 μm. n = 6 mice per group (f,g). Bars show mean ± SEM, Two-sided P values were determined by one-way ANOVA with multiple comparisons (b-e). Results represent four (b-e) and two (f,g) independent experiments. Source data

Finally, we sought to examine whether treatment with Mac1 cells could protect cardiac function after sepsis. The sorted TREM2^hi^ Mac1 and non-Mac1 cells from the hearts of CD45.1 mice were respectively transplanted into the pericardial space of CD45.2 mice immediately after CLP. The control animals were intrapericardially injected with the same volume of Matrigel (MG) (Fig. 7a). Flow cytometry analysis showed that the transplanted Mac1 and non-Mac1 cells can survive and persist in recipient hearts for at least 7 d after CLP (Extended Data Fig. 9a). The proportion of Mac1 cells in total CD45^+^ cells was significantly increased in the hearts of Mac1 cell-injected mice at the examined time points compared to MG-injected mice (Extended Data Fig. 9b,c). Similarly, the proportion of non-Mac1 cells in total CD45^+^ cells was also increased in the hearts of non-Mac1-injected mice at the examined time points compared to MG-injected mice (Extended Data Fig. 9d,e). Furthermore, Mac1 and non-Mac1 cells were labeled with CellTracker Orange (CMTMR) and then transferred into the pericardial cavity immediately after CLP. After 3 d, histological assay indicated that the transplanted cells were widely distributed in the myocardium (Extended Data Fig. 9f). In addition, we intrapericardially transferred the CMTMR-labeled Mac1 cells into MitoCard mice. Three days later, confocal and 3D images showed that injected cells engulfed cardiomyocyte-derived mitochondria (Extended Data Fig. 9g and Supplementary Video 6). These data collectively show that the transplanted Mac1 cells can enter the myocardium, survive in the heart of recipient mice for at least 7 d and engulf cardiomyocyte-derived mitochondria.

Compared to intrapericardial transplanted non-Mac1 cells or MG groups, hearts of septic mice that were transplanted with TREM2^hi^ Mac1 cells showed significantly enhanced cardiac function (Fig. 7b–e). Consistently, the transplantation with TREM2^hi^ Mac1 cells improved cardiac injury (Fig. 7f–i), inflammation (Fig. 7j,k), mitochondrial damage (Fig. 7l), increased ATP levels (Fig. 7m) and reduced lactate levels (Fig. 7n).

**Extended Data Fig. 10:**
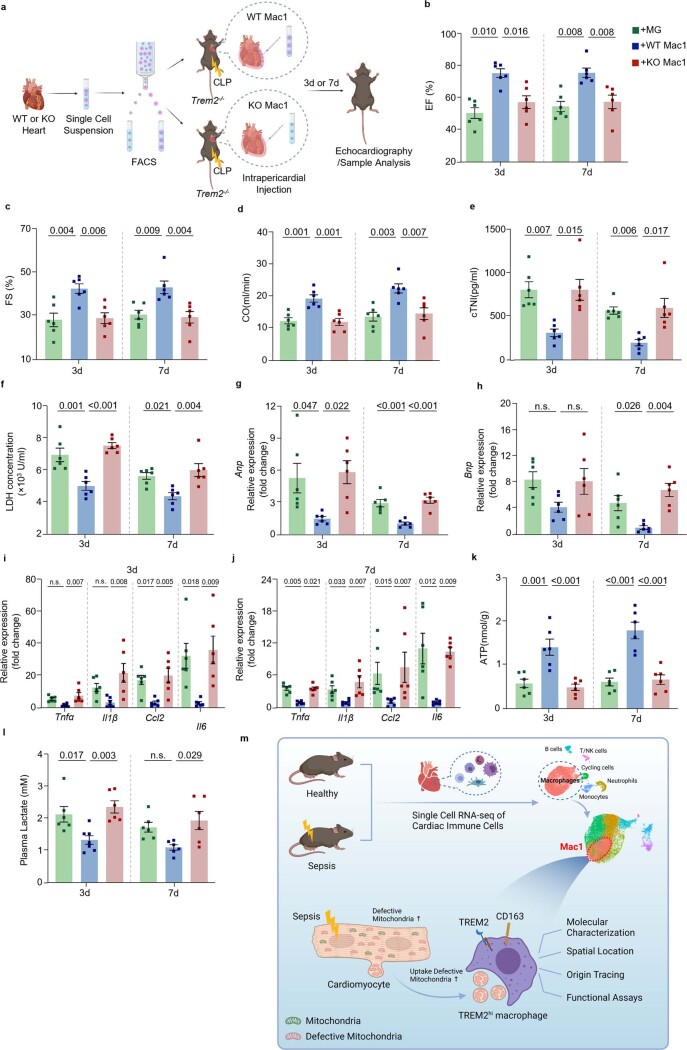
Intrapericardial administration of WT Mac1 cells protects SICM in *Trem2*^−/−^ mice, related to Fig. 7. a, Schematic illustration of Mac1 cells transplantation in Trem2^-/-^ mice. Mac1 (CD45^+^CD11b^+^F4/80^+^CD163^+^RETNLA^+^) cells isolated from WT and Trem2^-/-^ mice were mixed with Matrigel (MG), respectively, and then transplanted into the pericardial cavity of Trem2^-/-^ mice (2×10^5^ cells/animal) immediately after CLP. Control mice were injected MG only. Mice were analyzed 3 or 7 days after the transplantation. b-d, Graphs showing EF% (b), FS% (c) and CO (d) measured by echocardiography. e,f, Graphs showing the levels of cTnI (e) and LDH (f) in the serum. g,h, Graphs showing mRNA levels of *Anp* (g) and *Bnp* (h) in the heart tissue lysates. i,j, Graphs showing mRNA levels of *Tnfα*, *Il1β*, *Ccl2* and *Il6* in the heart tissue lysates at 3 (i) and 7 (j) days after CLP. k, Graph showing ATP content in heart tissue lysates. l, Graphs showing the levels of serum lactate. b-l, each symbol represents one animal (n = 6 mice for each treatment). All data are presented as mean ± SEM. Two-sided P values by Kruskal-Wallis with Dunn’s multiple comparison test, n.s., not significant. m, Schematic diagram showing TREM2^hi^ Mac1 phagocytoses cardiomyocyte-derived mitochondria protect the septic heart. scRNA-seq uncovers a cardiac macrophage subset that was highly expressing TREM2 and CD163; Sepsis stimulates the release of massively cardiomyocyte-derived mitochondria; TREM2^hi^ Mac1 cells scavenge defective mitochondria and protect the septic heart. Results represent two (b-l) independent experiments. Source data

Next, WT Mac1 cells and *Trem2*^−/−^ Mac1 cells were isolated from the hearts of WT or *Trem2*^−/−^ mice and intrapericardially transplanted (2 × 10^5^ cells per mouse) into the hearts of *Trem2*^−/−^ mice immediately after CLP (Extended Data Fig. 10a). Three and 7 d later, echocardiography revealed that transplantation with WT Mac1 cells significantly enhanced cardiac function of septic mice compared to *Trem2*^−/−^ mice injected with *Trem2*^−/−^ Mac1 cells or MG groups (Extended Data Fig. 10b–d). Consistently, the transplantation with WT Mac1 cells improved cardiac injury, inflammation (Extended Data Fig. 10e–j), enhanced ATP levels (Extended Data Fig. 10k) and reduced lactate levels (Extended Data Fig. 10l). Taken together, these results suggest that administration of TREM2^hi^ Mac1 cells may be a potential therapeutic approach for prevention and rescue of cardiac dysfunction in sepsis.

## Discussion

Patients with SICM have a high mortality rate^4,36,37^ and their septic hearts are associated with a high energetic metabolism and catastrophic mitochondrial damage^5,38^. Macrophages are involved in cardiac inflammation, efferocytosis, tissue remodeling and homeostasis^11,13,39^; however, the roles of distinct macrophage subsets in the septic heart have not been fully elucidated. In the present study, we investigated a cardiac macrophage subset in septic and steady-state hearts, which was featured by high expression of TREM2 and the ability of self-renewal, defined as TREM2^hi^ Mac1 cells. The high expression of TREM2 is essential for the function of Mac1 cells in scavenging defective mitochondria and protecting the septic heart (Extended Data Fig. 10m). In addition, intrapericardial injection of CD163^+^RETNLA^+^TREM2^hi^ Mac1 cells could prevent the cardiac dysfunction of septic mice.

Accumulating studies highlighted the heterogenous composition and tissue-specific changes of macrophages at steady state and different pathological settings such as myocardial infarction^8,11,19,22^. Our scRNA-seq data revealed the presence of five macrophage populations, Mac1–5, in the steady-state heart of mice, which were indicated by a previous study^19^. Mac1 and Mac2 subsets were the dominant cardiac macrophage subsets, which were featured by the expression of phagocytosis (*Trem2*) and antigen-presenting (*H2-Aa*)-related genes, respectively. The Mac3–5 subsets had low proportions and highly expressed genes related to IFN stimulation (*Irf7*), pathological inflammation (*CD72*) and chemokines (*Ccr2)*, respectively. We found that the Mac1 subset exhibited a dynamic change, decreased at 3 d and recovered at 7 d after CLP, which was well correlated with the deterioration and recovery of cardiac function in sepsis. Both Mac3 and Mac4 subsets were significantly increased along with exacerbated systemic inflammatory responses and bone marrow mobilization. A recent study showed that in a transverse aortic constriction model, CD72 was a key marker of a pro-inflammatory macrophage subset, in which many genes overlapped with Mac4 identified in our study^22^, suggesting that the CD72-expressed Mac4 subset is closely associated with inflammation and cardiac injury in SICM.

Regarding the origin, cardiac macrophages can be subdivided into embryo-derived resident CCR2^−^ subsets (resident) and hematopoietic-derived recruited CCR2^+^ subsets (circulating)^11–13^. CCR2^−^ subsets are replenished through local proliferation, mediate metabolic stability and promote cardiac recovery after injury^14,19^. In contrast, recruited CCR2^+^ subsets enhance inflammation and oxidative stress^40^. Dick et al. found that a TIMD4^+^LYVE1^+^MHC-II^lo^CCR2^−^ resident cardiac macrophage subset, which was maintained by self-renewal, inhibits adverse remodeling and promotes the recovery of cardiac function after myocardial infarction^14^. In line with this study, we identified a subset of CD163^+^RETNLA^+^ CRMs (Mac1 cells), which was remarkably decreased upon septic stress. Upon SICM progression, resident Mac1 cells displayed a self-renewal capability and presented a protective role in septic heart. A recent manuscript also showed that the number of cardiac macrophages was decreased 1 d after CLP and then increased to supra-normal levels by 28 d^41^. The recovery of cardiac macrophage numbers was dominated by local proliferation, whereas peripheral recruitment accounted for only 6.2%^41^. These findings suggest an essential role of self-renewing resident Mac1 cells in protecting cardiac function in sepsis.

As a transmembrane receptor of the immunoglobulin superfamily, the activation of TREM2 leads to DAP12 phosphorylation and consequently promotes cell proliferation and survival, modulates phagocytosis and counteracts inflammation^42,43^. We observed that the Mac1 subset features TREM2^hi^ and its proliferation was enhanced during sepsis. Furthermore, *Trem*2 deficiency did not affect the number of Mac1 cells in the healthy heart but significantly impaired the restoration of these macrophages in the septic heart. Our previous study showed that TREM2 was barely detectable in the liver, lung and spleen of steady-state mice, whereas TREM2 expression was significantly increased in these tissues after CLP and blockage of TREM2 exacerbated polymicrobial sepsis^44^, suggesting its protective effect against sepsis. The specific function of TREM2^+^ macrophages in other organs requires further investigation. Previous studies demonstrated that *Trem2* deficiency resulted in a decrease of macrophage proliferation in pathological conditions^45,46^ and induced cell cycle arrest^47^. Similarly, we noticed that *Trem*2 deficiency remarkably decreased the proliferation of Mac1 cells in SICM. Our findings suggest that TREM2 play essential roles in the recovery of SICM through the reconstitution of CD163^+^RETNLA^+^ Mac1 subpopulation.

Extracellular mitochondria and mtDNA can trigger inflammation and cause cardiac damage^29,30^. A recent study showed that CRMs cleared extruded mitochondrial debris to preserve the homeostasis of the long-lived cardiomyocytes and played protective function^14^. Our present findings indicated that dysfunctional mitochondria were remarkably increased in the extracellular space of myocardium, paralleled by cardiac dysfunction under septic stress. Consistent with previous results^41^, sepsis showed a very slight effect on the survival of cardiac cells. Mac1 cells exhibited a potent ability to remove cardiomyocyte-derived abnormal mitochondria in SICM. Moreover, *Trem*2 ablation downregulated the endocytic genes in CRMs and exacerbated the accumulation of defective mitochondria in the heart and cardiac dysfunction. In addition, intrapericardial administration of CD163^+^RETNLA^+^TREM2^hi^ macrophages could survive and engulf cardiomyocyte-derived defective mitochondria, which consequently protect against septic myocardial dysfunction. Our findings suggest that TREM2^hi^ Mac1 cells had a powerful endocytosis function and preserved cardiac homeostasis by eliminating defective mitochondria during sepsis.

In summary, our study has revealed a CD163^+^RETNLA^+^ macrophage subset (Mac1), which features high expression of TREM2. TREM2 is essential for self-renewal and remodeling of Mac1 cells in the septic heart. TREM2^hi^ Mac1 cells scavenge defective mitochondria and the transplantation of Mac1 cells contribute to the recovery of cardiac function during SICM. Our study highlights that maintaining the function of this subset by harnessing TREM2 could be a potential therapeutic strategy for cardiac diseases in clinic.

## Methods

### Mice

C57BL/6 WT mice were obtained from Shanghai SLAC Laboratory Animal Center. M. Colonna kindly provided *Trem2*^−/−^ mice from Washington University in St. Louis. M. Shang from Zhejiang University kindly provided αMHC^Cre^ mice. The following mice were purchased from Jackson Laboratory: Rosa26-stop-tdTomato, Cx3cr1^CreERT2–IRES–YFP^, mtD2^flox/+^ (B6;129S-Gt(ROSA)26Sor^tm1(CAG−COX8A/Dendra2)Dcc/J^) and B6;CD45.1 mice (B6.SJL-Ptprca Pepcb/BoyJ). αMHC^Cre^ mice were crossed with Rosa26-stop-tdTomato or mtD2^flox/flox^, to generate cardiomyocyte fluorescent reporter mice (referred to as Card^RED^ mice) or cardiomyocyte-specific mitochondria fluorescent reporter mice (referred to as MitoCard mice). Mice were housed in a specific-pathogen-free environment, with a 12 h light–dark cycle and the ambient temperature was maintained at 22 °C. Mice were fed irradiated chow (SZS9126, XIETONG BIO-ENGINEERING) and sterile water. Cages were autoclaved and changed once a week. Only male mice were used in the experiments. The animal experiments were approved by the Animal Care and Use Committee of Zhejiang University School of Medicine and performed according to institutional guidelines (reference nos. 2017442 and 2021858).

### Cecal ligation and puncture model

Anesthesia was administered to 8-week-old male mice with ketamine (50 mg kg^−1^) and xylazine (5 mg kg^−1^) intraperitoneally injected before abdominal depilation. Then mice were exposed to a 1.5-cm longitudinal cut in the lower quadrant of the abdomen and then the cecum was isolated. The distal three-quarters of the cecum was ligated with 4-0 silk sutures and two punctures were made in the ligated cecum with a 22-gauge needle, inducing sublethal CLP-induced sepsis. Then the cecum was placed back into the abdominal cavity after feces was extruded and the wound was sutured with a 4-0 silk suture. Each mouse was given 1 ml sterile saline after operation^48^. The survival rate was evaluated every 2 h for 7 d.

### Fate mapping

To fate-label cardiac macrophage, Cx3cr1^CreERT2–IRES–YFP^ mice were crossed to Rosa26-stop-tdTomato mice. At the age of 6 weeks, Cx3cr1^CreERT2^:*Rosa26*^tdTom^ mice were administrated with 4 mg tamoxifen (Meilunbio) per mouse in corn oil and again 48 h later. After 5 weeks, mice were killed for assays.

### Bone marrow chimeric mouse model

Bone marrow cells were obtained from femurs and tibias of 8-week-old donor male mice. First, bone marrow was flushed out with precooled PBS. After red blood cell lysis, a single-cell suspension was obtained through a 70-µm filter. A total of 1 × 10^7^ bone marrow cells were transferred into lethally irradiated (8 Gy) recipient male mice intravenously. The chimeric mice were given antibiotics through drinking water for 1 week. After 8 weeks, the model construction was completed.

### Generation and delivery of adeno-associated virus

Recombinant adeno-associated virus serotype 2/9 (AAV 2/9) vectors carrying mt-Keima with the cardiac-specific promoter cTnT were designed as previously described^14^ and manufactured by OBiO Technology. Each mouse was administered with a titer of 3 × 10^11^ AAV9-Tnnt2-mt-Keima virus via intra-cardiac injection. After 6 weeks, mice were used for CLP experiments.

### RNA extraction and RT–qPCR

Total RNA was obtained from mouse tissues and cells using TRIzol (Ambion). Complementary DNA samples were synthesized with PrimeScript RT reagent kit (Takara) and gene expression was analyzed with qPCR using SYBR premix Ex Taq (Takara) on Lightcycler 480 system (Roche). Primer sequences used are listed in Supplementary Table 1.

### Echocardiography

Echocardiography was administrated in mice under isoflurane anesthesia using a Vevo 2100 system (VisualSonics). Normal body temperature was maintained by a heating platform. Parasternal standard two-dimensional (2D) and M-mode short-axis view was used to measure the left ventricular (LV) internal dimensions at diastole (LVIDd) and systole (LVIDs), the LV internal volume at diastole (LVEDV) and systole (LVESV) and the heart rate (HR). The LV EF was acquired as ((LVEDV − LVESV) / LVEDV) × 100, the LV FS was acquired as ((LVIDd−LVIDs) / LVIDd) × 100 and CO was acquired as (LVEDV − LVESV) × HR. For diastolic dysfunction analysis, a 2D apical view was used by pulsed-wave Doppler. Early and late diastolic velocity peak waves (E and A, respectively) were detected and the E/A ratio was acquired. Image analysis was performed offline using Vevo LAB v.3.1.0 (VisualSonics).

### Immunofluorescence

Fresh hearts were embedded with optimal cutting temperature compound and cut into 10-μm sections. After drying in air for 1 h, slides were fixed in 4% paraformaldehyde for 20 min, permeabilized with 0.1% Triton X-100 in 0.1% sodium citrate for 15 min and blocked with blocking buffer (3% BSA + 5% FBS + 0.1% Tween 20) for 30 min at room temperature. Then tissue sections were incubated at 4 °C overnight with primary antibodies against rat anti-CD68 (1:250 dilution; Abcam), mouse anti-CD163 (1:100 dilution; Santa Cruz), rat anti-TREM2 (1:100 dilution; R&D), rabbit anti-Tom20 (1:200 dilution; Abcam), rat anti-LAMP1 (1:50 dilution, DSHB) and cTnI (1:250 dilution; Abcam). After being washed three times with PBST for 5 min each, slides were incubated with conjugated secondary antibodies at room temperature for 2 h, followed by washing three times with DPBS and sealing with DAPI-containing blocking agent. To analyze cardiomyocyte apoptosis, TUNEL staining was performed with a TUNEL kit according to the manufacturer’s instructions. Images were acquired with Olympus OSR confocal microscope (OLYMPUS IX83-FV 3000-OSR) or Zeiss LSM 880 (with fast AiryScan) and processed with by ImageJ Pro Plus v.6.0 software (Media Cybernetics).

To analyze the cardiomyocyte-derived vesicles containing mitochondria, samples from Card^RED^ mice were stained with rabbit anti-Tom20 primary antibody (1:250 dilution, Abcam) and Alexa fluor 488-conjugated donkey anti-rabbit secondary antibody (1:500 dilution, Invitrogen). Images were acquired using Zeiss LSM 880 (with AiryScan) at an interval of 0.38 μm per z-step (*z* = 7–8 μm) for 3D reconstructions. A 3D reconstruction analysis of above experiments was processed using Imaris software v.9.7 (Bitplane). After reconstructing cardiomyocytes, vesicles and mitochondria using a surface tool based on 0.2-mm detail and absolute intensity, we analyzed the distance between Tom20^+^tdTomato^+^ vesicles and cardiomyocytes to show that vesicles were outside of cardiomyocytes. Then we quantified Tom20^+^tdTomato^+^ vesicles on five random FOVs per sample.

Mt-Keima fluorescence was imaged in two channels by two sequential excitations (488 nm, green; 561 nm, red) using a Zeiss LSM 880 (Zeiss LSM 880 with fast AiryScan). Identical imaging settings were maintained to compare under different experimental conditions. All images were analyzed with ImageJ Pro Plus v.6.0 software (Media Cybernetics) to calculate the ratio of red fluorescent protein to green fluorescent protein area.

All antibodies applied are listed in Supplementary Table 2.

### Transmission electron microscopy

Collected heart samples were obtained from the anterior ventricular wall and cut into 2-mm^3^ cubes (2 × 1 × 1 mm). These samples were fixed in 2.5% glutaraldehyde (pH 7.2) for 2 d at room temperature. Then the cubes were embedded with epoxypropane resin following standard methods. The slices were observed and scanned using a Tecnai G2 Spirit 120 kV TEM (Thermo FEI) with a charge-coupled device camera.

### Semi-quantitative analysis of electron microscopic specimens

Myocardial mitochondria were observed using TEM and analyzed semi-quantitatively as described previously^49^. Five FOVs were randomly selected for photography at the same magnification and approximately 20 mitochondria in each field were randomly chosen for analysis. Each sample was analyzed for 100 mitochondria. Each mitochondrion was graded on a scale of 0–4 according to the degree of injury (higher scores indicated more severe injury).

### Detection of molecular markers in serum or heart

According to the manufacturer’s instructions, serum lactate levels were detected using a Lactic Acid Assay kit (Nanjing Jiancheng), serum LDH levels were tested using a Lactate Dehydrogenase Assay kit (Nanjing Jiancheng) and serum cTnI levels were determined using a mouse cTnI ELISA kit (Cloud-Clone). ATP content in the heart was measured using an ATP Assay kit (Beyotime) and serum ANP and NT-proBNP levels were determined using mouse ELISA kits (USCN Life Science). IL-1β, IL-6, TNF-α and CCL2 protein levels in heart tissue lysates were determined using mouse ELISA kits (NOVUS (IL-1β and IL-6) and MULTI SCIENCES (TNF-α and CCL2)).

### Isolation of cells from mouse heart tissue

Mice were anesthetized by isoflurane inhalation. Afterward, the hearts were perfused with 20 ml perfusion buffer (1× DPBS with 0.8 mM CaCl_2_) to remove peripheral blood from the chambers. Subsequently, atria and valves of the isolated heart were removed and ventricles were minced to ~1-mm cubes. The hearts were digested with 0.25 mg ml^−1^ of Liberase TL (Sigma-Aldrich), 20 μg ml^−1^ DNase I (Sinopharm Chemical Reagent) and 10 mM HEPES (Sigma-Aldrich) in serum-free DMEM (Gibco) at 37 °C for 15 min. Then, the tissue suspension was triturated using 1,000-μl micropipettes. The resulting cell suspension was obtained through a 70-μm filter to remove undigested tissue mass, washed with PBS containing 2% FBS and diluted in 1 ml HBSS. Then the cell suspension was spread on a 15% and 60% Percoll (Yeason) top layer and subjected to density gradient centrifugation at 400*g* for 20 min. The cell layer between the liquid surfaces was collected and washed with 10 ml DPBS to obtain single-cell suspensions for further experiments.

### Flow cytometry and fluorescence-activated cell sorting

For flow cytometric analysis, the single-cell suspensions were stained with relative antibodies at 4 °C for 30 min in a final volume of 200 μl. Cells were washed with DPBS, resuspended in a final volume of 400 μl and filtered through a 40-μm filter. Samples were collected on a BD Fortessa (BD Biosciences) by BD FACSDiva software v.8.0.1 and analyzed with FlowJo v.10.6.2 software (TreeStar). Details of the immunostaining and gating strategy of immune cells and cardiac macrophage subpopulations in the septic heart are shown in Extended Data Figs. 2d and 3b.

For measurement of mitochondrial function, L-929 fibroblasts were used as a control to evaluate treatment responsiveness compared to isolated vesicles. Oligomycin (Cell Signaling Technology, 5 μM) was employed to promote mitochondrial membrane hyperpolarization. Carbonyl cyanide-4 (trifluoromethoxy) phenylhydrazone (Sigma-Aldrich, 2 μM) was applied to uncouple oxidative phosphorylation in mitochondria and depolarize mitochondrial membranes. Both isolated vesicles and L-929 cells were administrated at 37 °C for 1.5 h. Then, cells or vesicles were stained in culture medium supplemented with 1× MitoNIR (Abcam), which is a fluorescent probe measuring mitochondrial membrane potential, for 20 min at 37 °C. Cells or vesicles were acquired by a BD Fortessa (BD Biosciences). MFI was analyzed using FlowJo software (TreeStar).

We assessed Ki67 expression by fixing and permeabilizing cells with a Foxp3 Transcription Factor Staining Buffer Set (Thermo Fisher Scientific) according to the manufacturer’s instructions and then staining cells with anti-Ki67 antibody as mentioned above.

For FACS sorting, the single-cell suspensions were stained with relative antibodies for 30 min at 4 °C and then were washed with DPBS. Pellets were resuspended with 200 μl DPBS. For scRNA-seq experiments, Calcein Blue AM (Thermo Fisher Scientific, 4 μg ml^−1^) and Vybrant DyeCycle Ruby (Thermo Fisher Scientific, 10 μM) were added in 200 μl solutions and were incubated for 10 min at 4 °C. After incubation, live nucleated CD45^+^ cells were obtained on a Beckman MoFlo Astrios EQ with a 100-μm nozzle (Beckman). For Mac1 transplantation experiments, TREM2^hi^ Mac1 (CD45^+^ CD11b^+^F4/80^+^CD163^+^RETNLA^+^) and non-Mac1 (CD45^+^CD11b^+^F4/80^+^ and non-CD163^+^RETNLA^+^) cardiac macrophages were sorted and collected separately for further experiments.

### Single-cell RNA-seq

Single cells were collected into RPMI 1640 containing 5% FBS. Trypan blue exclusion confirmed the cell viability. Sorted cells were counted and concentration adjusted to 700–1,200 cells μl^−1^. Then single-cell suspensions were loaded to a 10x Chromium to capture no more than 10,000 single cells by Chromium Single Cell 3′ Reagent kits v.3 (10x Genomics). The cells were partitioned into Gel Beads in the Chromium instrument, where cell lysis and barcoded reverse transcription of RNA occurred. DNA amplification and library construction were conducted. Libraries were sequenced on a Novaseq 6000 (Illumina) by LC-Bio Technology. The data were aligned to the GRCm38 reference genome using CellRanger v.4.0 (10x Genomics).

### Dimension reduction, clustering and differentially expressed gene analysis

The CellRanger output was loaded into Seurat (v.3.1.1) for unsupervised clustering. All genes expressed in fewer than one cell were removed. Cells expressing fewer than 200 and more than 6,000 genes, unique molecular identifier counts more than 40,000 and the percent of mitochondrial DNA (mtDNA) gene expression more than 20% were excluded. Mitochondrial genes were excluded from the expression matrix.

To visualize the data, we used the Seurat package for further analysis. First, we used the LogNormalize method of the Normalization function of the Seurat package to assess the expression value of genes. Second, we performed principal-component analysis on the normalized expression matrix with highly variable genes identified by FindVariableGenes function. Based on the top ten principal components, we obtained the unsupervised cell cluster result by weighted shared nearest neighbor graph-based clustering method. To detect cluster-specific genes, we identified the marker genes by the bimod (Likelihood-ratio test) of the FindAllMarkers function in Seurat. Compared to other clusters, the genes whose expression was more than 25% of the cells and average log (fold change) >0.26 in the target cluster, were defined as marker genes. Cell types were defined based on known markers. Cells expressing non-immune cell markers were excluded.

Cell re-clustering, marker gene visualization, DEG analysis, GO enrichment analysis and other bioinformatics analyses were performed with the OmicStudio tools (https://www.omicstudio.cn/tool). DEGs were identified by the bimod with default parameters through the FindAllMarkers function under the following criteria: (1) log (fold change) >0.26; (2) *P* value <0.01; and (3) min.pct > 0.1.

Monocytes and macrophages from the four WT samples are shown in Fig. 2a–d. Fig. 4e,f shows *Trem2*^−/−^ samples and WT littermate control samples 7 d after CLP, aggregated and analyzed as described above to compare the impact of *Trem2*^−/−^ on the monocyte-macrophage compartment.

### Single-cell trajectory analysis

For single-cell trajectory analysis, we used Monocle v.2.4.0 to investigate developmental trajectories between macrophage/monocyte subsets^19,27^. scRNA-seq data were scaled, normalized and clustered by the Seurat tool, then downsampled to 1,500 cells and loaded into a Monocle object. The DifferentialGeneTest function was used to infer DEGs from each cluster and we used the top 800 genes with the lowest *q* value to sequence the cells in pseudotime analysis. The developmental trajectory was plotted by plot_cell_trajectory command. The ‘root_state’ (the starting point) of the trajectory was defined as the terminal with lower expression of monocyte genes. After the cell trajectories were constructed, DEGs along the pseudotime were detected by the DifferentialGeneTest function. Changes in the top 50 genes through pseudotime with the lowest *q* values were visualized by a heat map using the plot_pseudotime_heatmap function. Plot_genes_in_pseudotime function was used to show the dynamic trend of the selected significant gene expression levels.

### Preparation of cardiac vesicles for flow cytometry and sorting

Card^RED^ mice hearts were cut into small pieces and digested in DPBS with 0.25 mg ml^−1^ of Liberase TL (Sigma-Aldrich), 20 μg ml^−1^ DNase I (Sinopharm Chemical Reagent) and 10 mM HEPES (Sigma-Aldrich) for 15 min at 37 °C. Then, tissue suspensions were acquired by gentle pipetting. We serially centrifuged these suspensions at 50*g* and 300*g* and discarded the pellet. The supernatant was then centrifuged at 1,000*g* and washed with DPBS. Pellets were resuspended with 200 μl DPBS. We used the endogenous expression of tdTomato, CD31 and Vybrant DyeCycle Ruby (Thermo Fisher Scientific, 10 μM) to define cardiac vesicles. The gating strategy for cardiac vesicles is shown in Extended Data Fig. 6c. The material was sorted in a Beckman MoFlo Astrios EQ (Beckman) with a 100-μm nozzle.

### Intrapericardial injection of sorted cardiac macrophages

FACS-sorted cells were washed with DPBS and quantified. Cardiac macrophages (2 × 10^5^ cells) were resuspended in 25 μl MG (Corning). After anesthetizing and intubating the recipient mice, the mouse chest was opened and MG containing cardiac macrophages was injected into the pericardial cavity using a 30-gauge needle. The control animals were only intrapericardially injected with 25 μl MG.

For cell survival and distribution experiments, the sorted Mac1 and non-Mac1 cells were stained with CMTMR (50 nM) at 37 °C for 20 min and then washed with DPBS. Labeled macrophages (2 × 10^5^ cells) were resuspended in 25 μl MG and then transplanted into the pericardial cavity immediately after CLP. Three days later the mice were killed. Heart samples were collected and cryosections were made. Images were acquired using a Zeiss LSM 880 (with fast AiryScan) according to standard procedures and analyzed with ImageJ Pro Plus v.6.0 software.

### Protein array assay

Total protein was isolated from the mouse heart using RIPA (Applygen) and the supernatant was analyzed by a glass-based and sandwich-based antibody microarray to measure 20 cytokines quantitatively (RayBiotech). Each cytokine was analyzed in quadruplicate per array. Then, 100 µl supernatant were loaded to each well, incubated overnight at 4 °C and then extensively washed. A biotin-labeled detection antibody was incubated for 2 h followed by the application of AlexaFluor 555-conjugated streptavidin at room temperature for 1 h. The slides were analyzed by an InnoScan 300 Scanner (Innopsys) with 532 nm excitation and 635 nm emission. We obtained raw data (images) from the scanner and integrated spot intensities (tab-delimited .txt file) using Mapix v.7.3.1 software. Data visualization was obtained by Q-Analyzer Software (RayBiotech). All raw data were log_2_-converted for statistical analysis.

### Statistical analysis

All statistical analyses were performed using SPSS v.21.0 (IBM) or Prism v.8.0 (GraphPad Software). For comparison between two groups, normally distributed data were evaluated by the unpaired two-tailed Student’s *t*-test and data without a normal distribution were evaluated by the Mann–Whitney *U*-test. For more than two groups, normally distributed data were evaluated by one-way or two-way ANOVA, followed by Games-Howell’s, Sidak’s or Tukey’s multiple comparisons test and data without a normal distribution were evaluated by the Kruskal–Wallis with Dunn’s multiple comparison test. Correlations were analyzed by Pearson’s correlation coefficient. The survival rate was analyzed by Kaplan–Meier analysis and log-rank test. Data are presented as mean ± s.e.m. or median with interquartile range

### Reporting summary

Further information on research design is available in the Nature Portfolio Reporting Summary linked to this article.

## Supplementary information

Supplementary Information Supplementary Tables 1–3 and legends for Supplementary Videos 1–6.

Reporting Summary

Supplementary Video 1 Sepsis induces the release of cardiomyocyte-derived exophers. 3D reconstruction of heart slices from Card^RED^ mice show the presence of mitochondria (Tom20, cyan) in cardiomyocyte-derived exophers (red) and the accumulation of Tomato^+^ exophers colocalized with Tom20 in septic hearts.

Supplementary Video 2 Mac1 cells take up cardiac exophers containing mitochondria in SICM. 3D reconstruction of the heart slices from Card^RED^ mice. Mac1 cells (TREM2, green) phagocytosed cardiomyocyte-derived exophers (red). Exophers in Mac1 cells included mitochondria (Tom20, white).

Supplementary Video 3 Cardiomyocyte-derived mitochondria transfer to Mac1 cells. 3D reconstruction of the heart slices from MitoCard mice. Mac1 cells (TREM2, red) took up cardiomyocyte-derived mitochondria (mtDendra2, green).

Supplementary Video 4 Cardiomyocyte-derived mitochondria processed with LAMP1^+^ phagolysosomes in Mac1 cells. 3D reconstruction of the heart slices from MitoCard mice. Cardiomyocyte-derived mitochondria (mtDendra2, green) phagocytosed by Mac1 cells (TREM2, red) partially localized in lysosomes (LAMP1, white).

Supplementary Video 5 *Trem2* deficiency impairs the uptake of cardiomyocyte-derived mitochondria by Mac1 subset in septic heart*.* Part 1: Schematic illustration of the cardiomyocyte mitochondria labeled with AAV9-Tnnt2-mt-Keima virus. Keima-tagged mitochondria were indicated by different fluorescence in neutral (Keima 458nm, green) and acidic (Keima 561nm, red) environments. Part 2: 3D reconstruction showed that TREM2^+^ macrophages (green) took up cardiomyocyte-derived mitochondria (mtKeima-458, cyan) and some mitochondria in an acidic environment (mtKeima-561, red) in the hearts of AAV9-Tnnt2-mt-Keima-infected mice. Part 3: CD163^+^ macrophages (green) took up cardiomyocyte-derived mitochondria (mtKeima-458, cyan; mtKeima-561, red) in hearts of WT and *Trem2*^−*/*−^ mice infected with AAV9-Tnnt2-mt-Keima.

Supplementary Video 6 Transplanted Mac1 cells engulf cardiomyocyte-derived mitochondria in SICM. 3D reconstruction of the heart slices from MitoCard mice. Injected Mac1 cells (CMTMR, red) phagocytosed cardiomyocyte-derived mitochondria (mtDendra2, green).

## Data Availability

scRNA-seq data for this study have been deposited at the Gene Expression Omnibus under accession code GSE190856. All data and materials are available in the paper and the supplementary information. Source data are provided with this paper.

## Source data

Source Data Fig. 1 Single-cell RNA-seq data and statistical source data.
Source Data Fig. 2 Single-cell RNA-seq data and statistical source data.
Source Data Fig. 3 Statistical source data.
Source Data Fig. 4 Statistical source data.
Source Data Fig. 5 Statistical source data.
Source Data Fig. 6 Statistical source data.
Source Data Fig. 7 Statistical source data.
Source Data Extended Data Fig. 1 Statistical source data.
Source Data Extended Data Fig. 2 Statistical source data.
Source Data Extended Data Fig. 3 Statistical source data.
Source Data Extended Data Fig. 4 Statistical source data.
Source Data Extended Data Fig. 5 Single-cell RNA-seq data and statistical source data.
Source Data Extended Data Fig. 6 Statistical source data.
Source Data Extended Data Fig. 7 Single-cell RNA-seq data and statistical source data.
Source Data Extended Data Fig. 8 Statistical source data.
Source Data Extended Data Fig. 9 Statistical source data.
Source Data Extended Data Fig. 10 Statistical source data.
