# Supplementary material for: TREM2hi resident macrophages protect the septic heart by maintaining cardiomyocyte homeostasis
Source: Nat Metab. 2023 Jan 12;5(1):129–46. doi: 10.1038/s42255-022-00715-5 (PMC9886554; doi:10.1038/s42255-022-00715-5)
Supplement: Supplementary file 1 — Supplementary Tables 1–3 and legends for Supplementary Videos 1–6. [file 42255_2022_715_MOESM1_ESM.pdf]

nature metabolism

Article

https://doi.org/10.1038/s42255-022-00715-5

TREM2^{hi} resident macrophages protect the septic heart by maintaining cardiomyocyte homeostasis

In the format provided by the authors and unedited

The file includes:

Supplementary Table 1: List of primers used for RT-qPCR

Supplementary Table 2: List of Antibodies

Supplementary Table 3: List of chemicals and assays

Video S1. Sepsis induces the release of massively cardiomyocyte-derived exophers.

Video S2. Mac1 cells take up cardiac exophers containing mitochondria in SICM.

Video S3. Cardiomyocyte-derived mitochondria transfer to Mac1 cells.

Video S4. Cardiomyocyte-derived mitochondria processed with LAMP1$^+$ phagolysosomes in Mac1 cells.

Video S5. *Trem2* deficiency impairs the uptake of cardiomyocyte-derived mitochondria by Mac1 subset in septic heart.

Video S6. Injected Mac1 cells take up mitochondria form cardiomyocytes in SICM.

Supplementary Table 1: List of primers used for RT-qPCR.

All primers are purchased from Sangon Biotech.

**Gene**	**Forward Primer (5'→3')**	**Reversed primer (5'→3')**
β-actin	CAGCTTCTTTGCAGCTCCTT	GCAGCGATATCGTCATCCA
Trem2	TGGGTCCCCGAGGAGTCATC	AAACTTGCTCAGGAGAACGC
Anp	CACAGATCTGATGGATTTCAAGA	CCTCATCTTCTACCGGCATC
Bnp	GTCAGTCGTTTGGGCTGTAAC	AGACCCAGGCAGAGTCAGAA
αMhc	TGCACTACGGAAACATGAAGTT	CGATGGAATAGTACACTTGCTGT
βMhc	GATGTTTTTGTGCCCGATGA	CAGTCACCGTCTTGCCATTCT
Il1b	CTTCCCCAGGGCATGTTAAG	ACCCTGAGCGACCTGTCTTG
Il6	TTCCATCCAGTTGCCTTCTTG	TTGGGAGTGGTATCCTCTGTGA
Ccl2	TAAAAACCTGGATCGGAACCAAA	GCATTAGCTTCAGATTTACGGGT
Tnfα	ATGGCCTCCCTCTCATCAGT	CTTGGTGGTTTGCTACGACG

Supplementary Table 2: List of Antibodies.

**Antibodies**	**Source**	**Catalog**	**dilution**
AF700 Rat anti-Mouse CD45 (30-F11)	BD Biosciences	560510	1 : 200
PE-Cy5 Rat anti-Mouse CD45R/B220 (RA3-6B2)	BD Biosciences	553091	1 : 200
BV510 Hamster anti-Mouse CD3e (145-2C11)	BD Biosciences	563024	1 : 200
BV786 Mouse anti-Mouse NK-1.1 (PK136)	BD Biosciences	740853	1 : 200
BV711 Rat anti-Mouse CD11b (M1/70)	BD Biosciences	563168	1 : 200
FITC Rat anti-Mouse Ly-6G (1A8-Ly6g)	Thermo Fisher Scientific	11-9668-82	1 : 200
PE Rat anti-Mouse F4/80 (BM8)	BioLegend	123110	1 : 200
BV650 Rat anti-Mouse I-A/I-E (M5/114.15.2)	BD Biosciences	563415	1 : 200
BV605 Hamster anti-Mouse CD11c (N418)	BD Biosciences	744179	1 : 200
BV421 Rat anti-Mouse Ly-6C (HK1.4)	BioLegend	128031	1 : 200
PerCP-Cyanine5.5 Rat anti-Mouse/Human CD11b (M1/70)	BioLegend	101228	1 : 200
PE-Cyanine7 Rat anti-Mouse F4/80 (BM8)	Thermo Fisher Scientific	25-4801-82	1 : 200

Super Bright 600 Rat anti-Mouse CD163 (TNKUPJ)	Thermo Fisher Scientific	63-1631-82	1 : 200
PerCP-Cy5.5 Mouse anti-Mouse CD45.1 (A20)	BD Biosciences	560580	1 : 200
BV605 Mouse anti-mouse CD45.2 (104)	BD Biosciences	563051	1 : 200
Biotinylated Rabbit anti-Murine RELMα	PeproTech	500-P214BT	1 : 200
BV421 Mouse anti-Ki-67 (B56)	BD Biosciences	565929	1 : 200
BV421 Mouse IgG1, k Isotype Control (X40)	BD Biosciences	562438	1 : 200
Rat anti-Mouse CD68 (FA-11)	Abcam	ab53444	1:250
Mouse anti-Mouse CD163 (ED2)	Santa Cruz Biotechnology	sc-58965	1:100
Goat anti mouse TREM2 Antibody(polyclonal)	Abcam	ab95470	1:100
Rat anti Human/Mouse TREM2 (237920)	R and D Systems	MAB17291	1:100
Rabbit anti-Mouse TOMM20 (ERP15581-54)	Abcam	ab186735	1:200/1:250
Rabbit anti-Cardiac Troponin I (polyclonal)	Abcam	ab47003	1:250
Rat anti-Mouse LAMP1	DSHB	P11438	1:50

Alexa Fluor 594 Donkey anti-Rat IgG (H+L) Highly Cross-Adsorbed Secondary Antibody	Thermo Fisher Scientific	A21209	1:500
Alexa Fluor 488 Donkey anti-Mouse IgG (H+L) Highly Cross-Adsorbed Secondary Antibody	Thermo Fisher Scientific	A21202	1:500
Alexa Fluor 488 Donkey anti-Rabbit IgG (H+L) Highly Cross-Adsorbed Secondary Antibody	Thermo Fisher Scientific	A21206	1:500
Alexa Fluor Plus 647 Donkey anti-Rat IgG (H+L) Highly Cross-Adsorbed Secondary Antibody	Thermo Fisher Scientific	A48272	1:500
Alexa Fluor 594 Goat anti-Mouse IgG (H+L) Cross-Adsorbed Secondary Antibody	Thermo Fisher Scientific	A11005	1:500

Supplementary Table 3: List of chemicals and assays.

REAGENT	Source	Catalog
FITC TUNEL Assay Kit	Abcam	ab66108
MitoTracker™ Green FM	Thermo Fisher Scientific	M7514
CytoTracker Orange (CMTMR)	Thermo Fisher Scientific	C2927
NIR Mitochondrial Membrane Potential Assay Kit	Abcam	ab112149
Liberase™ TL Research Grade	Sigma-Aldrich	5401020001
Percoll	Yeason	40501ES60
Dnase I	Sinopharm Chemical Reagent Co.	64002860
HEPES	Sigma-Aldrich	V900477
Tamoxifen Citrate	Meilunbio	MB1233
DAPI-Fluoromount-G	Southern Biotech	0100-20
Corning® Matrigel® Basement Membrane Matrix, LDEV-free, 5 mL	Corning	356234
Mouse Interleukin Array Q1	Ray Biotech	QAM-INT-1
Lactic Acid assay kit	Nanjing Jiancheng Bioengineering Institute	A019-2-1
Lactate dehydrogenase assay kit	Nanjing Jiancheng Bioengineering Institute	A020-2-2
Mouse IL-1 beta Valukine ELISA	Novus	VAL601
Mouse IL-6 Valukine ELISA Kit	Novus	VAL604

Mouse TNF-a ELISA Kit	MULTI SCIENCES	EK282
Mouse CCL2/MCP-1 ELISA Kit	MULTI SCIENCES	EK287
ELISA Kit for Atrial Natriuretic Peptide (ANP)	USCN Life Science	SEA225Mu
ELISA Kit for N-Terminal Pro-Brain Natriuretic Peptide (NT-ProBNP)	USCN Life Science	SEA485Mu
ELISA Kit for Cardiac Troponin I (cTnI)	USCN Life Science	SEA478Mu
ATP Assay Kit	Beyotime	S0026
UltraComp eBeads™ Compensation Beads	Thermo Fisher Scientific	01-2222-42
eBioscience™ Foxp3 / Transcription Factor Staining Buffer Set	Thermo Fisher Scientific	00-5523-00
Chromium Next GEM Single Cell 3' GEM, Library & Gel Bead Kit v3.1, 16 rxns	10x Genomics	PN-1000121
Chromium Next GEM Chip G Single Cell Kit, 48rxns	10x Genomics	PN-1000120
TRIzol™ Reagent	Ambion	15596018
PrimeScript™ RT reagent Kit (Perfect Real Time)	Takara	RR037A
TB Green® Premix Ex Taq™ (Tli RNaseH Plus)	Takara	RR420A
eBioscience™ Calcein Violet 450 AM Viability Dye	Thermo Fisher Scientific	65-0854-39

Vybrant™ DyeCycle™ Ruby Stain	Thermo Fisher Scientific	V10309

Supplementary Video 1. Sepsis induces the release of massively cardiomyocyte-derived exophers.

3D reconstruction of the heart slices from Card^{RED} mice showed the presence of mitochondria (Tom20, cyan) in cardiomyocyte-derived exophers (red) and the accumulation of Tomato^{+} exophers co-localized with Tom20 in septic hearts.

Supplementary Video 2. Mac1 cells take up cardiac exophers containing mitochondria in SICM.

3D reconstruction of the heart slices from Card^{RED} mice. Mac1 cells (TREM2, green) phagocytosed cardiomyocyte-derived exophers (red). Exophers in Mac1 cells included mitochondria (Tom20, white).

Supplementary Video 3. Cardiomyocyte-derived mitochondria transfer to Mac1 cells.

3D reconstruction of the heart slices from MitoCard mice. Mac1 cells (TREM2, red) took up cardiomyocyte-derived mitochondria (mtDendra2, green).

Supplementary Video 4. Cardiomyocyte-derived mitochondria processed with LAMP1^{+} phagolysosomes in Mac1 cells.

3D reconstruction of the heart slices from MitoCard mice. Cardiomyocyte-derived mitochondria (mtDendra2, green) phagocytosed by Mac1 cells (TREM2, red) partially localized in lysosomes (LAMP1, white).

Supplementary Video 5. *Trem2* deficiency impairs the uptake of cardiomyocyte-derived mitochondria by Mac1 subset in septic heart.

Part 1: Schematic illustration of the cardiomyocyte mitochondria labeled with AAV9-Tnnt2-mt-Keima virus. Keima-tagged mitochondria were indicated by different fluorescence in neutral (Keima 458nm, green) and acidic (Keima 561nm, red) environments. Part 2: 3D reconstruction showed that TREM2$^+$ macrophages (green) took up cardiomyocyte-derived mitochondria (mtKeima-458, cyan) and some mitochondria in an acidic environment (mtKeima-561, red) in the hearts of AAV9-Tnnt2-mt-Keima infected mice. Part 3: CD163$^+$ macrophages (green) took up cardiomyocyte-derived mitochondria (mtKeima-458, cyan; mtKeima-561, red) in hearts of WT and *Trem2*$^{-/-}$ mice infected with AAV9-Tnnt2-mt-Keima.

Supplementary Video 6. Transplanted Mac1 cells engulf cardiomyocyte-derived mitochondria in SICM.

3D reconstruction of the heart slices from MitoCard mice. Injected Mac1 cells (CMTMR, red) phagocytosed cardiomyocyte-derived mitochondria (mtDendra2, green).
