# Supplementary material for: TREM2hi resident macrophages protect the septic heart by maintaining cardiomyocyte homeostasis
Source: Nat Metab. 2023 Jan 12;5(1):129–46. doi: 10.1038/s42255-022-00715-5 (PMC9886554; doi:10.1038/s42255-022-00715-5)
Supplement: Supplementary file 2 — Reporting Summary [file 42255_2022_715_MOESM2_ESM.pdf]

nature portfolio

Corresponding author(s): Qixing Chen; Xuekun Li; Xiangming Fang

Last updated by author(s): Nov 17, 2022

Reporting Summary

Nature Portfolio wishes to improve the reproducibility of the work that we publish. This form provides structure for consistency and transparency in reporting. For further information on Nature Portfolio policies, see our Editorial Policies and the Editorial Policy Checklist.

Statistics

For all statistical analyses, confirm that the following items are present in the figure legend, table legend, main text, or Methods section.

n/a	Confirmed	
[ ]	[x]	The exact sample size (*n*) for each experimental group/condition, given as a discrete number and unit of measurement
[ ]	[x]	A statement on whether measurements were taken from distinct samples or whether the same sample was measured repeatedly
[ ]	[x]	The statistical test(s) used AND whether they are one- or two-sided<br>*Only common tests should be described solely by name; describe more complex techniques in the Methods section.*
[x]	[ ]	A description of all covariates tested
[ ]	[x]	A description of any assumptions or corrections, such as tests of normality and adjustment for multiple comparisons
[ ]	[x]	A full description of the statistical parameters including central tendency (e.g. means) or other basic estimates (e.g. regression coefficient) AND variation (e.g. standard deviation) or associated estimates of uncertainty (e.g. confidence intervals)
[ ]	[x]	For null hypothesis testing, the test statistic (e.g. F, t, r) with confidence intervals, effect sizes, degrees of freedom and P value noted<br>*Give P values as exact values whenever suitable.*
[x]	[ ]	For Bayesian analysis, information on the choice of priors and Markov chain Monte Carlo settings
[x]	[ ]	For hierarchical and complex designs, identification of the appropriate level for tests and full reporting of outcomes
[x]	[ ]	Estimates of effect sizes (e.g. Cohen's d, Pearson's r), indicating how they were calculated

Our web collection on statistics for biologists contains articles on many of the points above.

Software and code

Policy information about availability of computer code

Data collection	Single-cell RNA sequencing were obtained using 10x Genomics (10x Genomics, USA).<br>TEM images were obtained with Tecnai G2 spirit 120kV transmission electron microscope (Thermo FEI, Czekh).<br>Fluorescent histological images were obtained with a OLYMPUS IX83-FV3000-OSR Confocal microscope (OLYMPUS, Japan) using the FV3000-FV31S-SW V2.1 and a LSM 880 with fast AiryScan Confocal microscope using Zeiss ZEN 2 (Zeiss, German).<br>Ultrasonographic images were obtained and analyzed by VisualSonics Vevo2100 (Visualsonics, Canada).<br>qPCR was carried out using LightCycler480 Software 1.5.0 (Roche, USA).<br>Flow cytometry were obtained with a BD Fortessa multicolor flow cytometer using BD FACSDiva Software v8.0.1 (BD, USA).<br>Protein array assay using Mapix 7.3.1 Software(INNOPSYS, France)
Data analysis	GraphPad Prism 8 software (GraphPad Software, USA) and IBM SPSS Statistics 21.0 (IBM, USA) were used for statistics analysis.<br>Fluorescent histological images were analyzed using ImageJ Plus Pro6.0 (Media Cybernetics, USA), Imaris software Version 9.7 (Bitplane, Switzerland).<br>FlowJo 10.6.2 (BD, USA) was used to analyze flow cytometry data.<br>Vevo LAB 3.1.0 (Visualsonics, Canada) was used to analyze Ultrasonographic data.<br>Single-cell RNA sequencing data was analyzed using Cell Ranger v4.0.0, Seurat 3.1.1, Monocle 2.4.0, OmicStudio tools (https://www.omicstudio.cn/tool), R 4.0.2, R studio<br>Q-Analyzer Software, SkanIt RE for Varioskan Flash 2.4.5, .

For manuscripts utilizing custom algorithms or software that are central to the research but not yet described in published literature, software must be made available to editors and reviewers. We strongly encourage code deposition in a community repository (e.g. GitHub). See the Nature Portfolio guidelines for submitting code & software for further information.

Data

Policy information about availability of data

All manuscripts must include a data availability statement. This statement should provide the following information, where applicable:

- Accession codes, unique identifiers, or web links for publicly available datasets
- A description of any restrictions on data availability
- For clinical datasets or third party data, please ensure that the statement adheres to our policy

Single-cell RNA-seq data for this study have been deposited at NIH GEO: GSE190856. All data and materials are available in the paper and the supplementary information. Source data are provided in this paper.

Field-specific reporting

Please select the one below that is the best fit for your research. If you are not sure, read the appropriate sections before making your selection.

- [x] Life sciences
- [ ] Behavioural & social sciences
- [ ] Ecological, evolutionary & environmental sciences

For a reference copy of the document with all sections, see nature.com/documents/nr-reporting-summary-flat.pdf

Life sciences study design

All studies must disclose on these points even when the disclosure is negative.

Sample size	Sample size for RNA-seq experiments were based on published studies in the field (e.g. Sarah A et al, Nature Immunology, 2019; Dawn M et al, Nature Medicine, 2019). Other sample size was based on the type of measurements made and experience. In vivo experiment, n=3-30 mice in each group, and the specific number is indicated in the figure legends; For ex vivo and in vitro experiments,, n=3-4 biologically independent. The sample size is not predicted by statistical method.
Data exclusions	We removed single cells with low quality in the scRNA-seq and mice with failed cecum ligation and puncture. We did not exclude any other data from the dataset.
Replication	Single-cell RNA sequencing (scRNA-seq) was performed on pooled 3-4 samples at each time point. Other experiments were independently performed at least three times, all experiments at replication were successful.
Randomization	All mice were randomly assigned into control and experimental groups. For ex vivo and in vitro experiments, the sample is randomized
Blinding	Technicians were blinded to genotype through random numbering of groups during experiments. Data analysis was blinding for all technicians and performed by a second technician.

Reporting for specific materials, systems and methods

We require information from authors about some types of materials, experimental systems and methods used in many studies. Here, indicate whether each material, system or method listed is relevant to your study. If you are not sure if a list item applies to your research, read the appropriate section before selecting a response.

Materials & experimental systems

n/a	Involved in the study	
[ ]	[x]	Antibodies
[ ]	[x]	Eukaryotic cell lines
[x]	[ ]	Palaeontology and archaeology
[ ]	[x]	Animals and other organisms
[x]	[ ]	Human research participants
[x]	[ ]	Clinical data
[x]	[ ]	Dual use research of concern

Methods

n/a	Involved in the study	
[x]	[ ]	ChIP-seq
[ ]	[x]	Flow cytometry
[x]	[ ]	MRI-based neuroimaging

Antibodies

Antibodies used	Primary antibodies:<br>Rat anti-Mouse CD45 (30-F11), BD Biosciences, Cat# 560510, 1:200<br>Rat anti-Mouse CD45R/B220 (RA3-6B2), BD Biosciences, Cat# 553091, 1:200<br>Hamster anti-Mouse CD3e (145-2C11), BD Biosciences, Cat# 563024, 1:200<br>Mouse anti-Mouse NK-1.1 (PK136), BD Biosciences, Cat# 740853, 1:200<br>Rat anti-Mouse CD11b(M1/70), BD Biosciences, Cat# 563168, 1:200

Rat anti-Mouse Ly-6G Antibody(1A8-Ly6g), Thermo Fisher Scientific, Cat# 11-9668-82, 1:200
Rat anti-mouse F4/80 Antibody(BM8), BioLegend, Cat# 123110, 1:200
Rat anti-Mouse I-A/I-E(M5/114.15.2), BD Biosciences, Cat# 563415, 1:200
Hamster anti-Mouse CD11c(N418), BD Biosciences, Cat# 744179, 1:200
Rat anti-mouse Ly-6C Antibody(HK1.4), BioLegend, Cat# 128031, 1:200
Rat anti-mouse/human CD11b antibody(M1/70), BioLegend, Cat# 101228, 1:200
Rat anti-mouse F4/80 Antibody(BM8),Thermo Fisher Scientific, Cat# 25-4801-82, 1:200
Rat anti-mouse CD163 Antibody(TNKUPJ), Thermo Fisher Scientific, Cat# 63-1631-82, 1:200
Rabbit anti-Murine RELMα Antibody(polyclonal), PeproTech, Cat# 500-P214bt-50ug, 100ng/mL
Mouse anti-Ki-67 Antibody(B56), BD Biosciences, Cat# 565929, 1:50
Rat anti mouse CD68 Antibody(FA-11), Abcam, Cat# ab53444, 1:250
Rat anti Human/Mouse TREM2 Antibody(237920), R and D Systems, Cat# MAB17291, 1:100
Goat anti mouse TREM2 Antibody(polyclonal), Abcam, Cat# ab95470, 1:100
Mouse anti-mouse CD45.1(A20), BD Biosciences, Cat# 560580, 1:200
Mouse anti-mouse CD45.2(104), BD Biosciences, Cat# 563051, 1:200
BV421 Mouse IgG1, k Isotype Control (X40), BD Biosciences, Cat# 562438, 1:200
Mouse anti-Mouse CD163 (ED2), Santa Cruz Biotechnology, Cat# sc-58965, 1:100
Rabbit anti-Mouse TOMM20 (ERP15581-54), Abcam, Cat# ab186735, 1:200/1:250
Rabbit anti-Cardiac Troponin I (polyclonal), Abcam, Cat# ab47003, 1:250
Rat anti-Mouse LAMP1, DSHB, Cat# P11438, 1:50

Secondary antibodies
Alexa Fluor 594 Donkey anti-Rat IgG (H+L) Highly Cross-Adsorbed Secondary Antibody, Thermo Fisher Scientific, Cat# A21209, 1:500
Alexa Fluor 488 Donkey anti-Mouse IgG (H+L) ReadyProbes™ Secondary Antibody, Thermo Fisher Scientific Cat# A21202, 1:500
Alexa Fluor 594 Goat anti-Mouse IgG (H+L) Cross-Adsorbed Secondary Antibody, Thermo Fisher Scientific Cat# A11005, 1:500
Alexa Fluor 488 Donkey anti-Rabbit IgG (H+L) Highly Cross-Adsorbed Secondary Antibody, Thermo Fisher Scientific Cat# A21206, 1:500
Alexa Fluor 647 Donkey anti-Rat IgG (H+L) Highly Cross-Adsorbed Secondary Antibody, Thermo Fisher Scientific Cat# A48272, 1:500

Validation

All antibodies were commercially available and validated for the species and application by the company, as well as other researchers.
Rat anti-Mouse CD45 (30-F11) for flow cytometry, https://www.bdbiosciences.com/en-us/products/reagents/flow-cytometry-reagents/research-reagents/single-color-antibodies-ruo/alexa-fluor-700-rat-anti-mouse-cd45.560510
Rat anti-Mouse CD45R/B220 (RA3-6B2) for flow cytometry, https://www.bdbiosciences.com/zh-cn/products/reagents/flow-cytometry-reagents/research-reagents/single-color-antibodies-ruo/pe-cy-5-rat-anti-mouse-cd45r-b220.553091
Hamster anti-Mouse CD3e (145-2C11) for flow cytometry, https://www.bdbiosciences.com/zh-cn/products/reagents/flow-cytometry-reagents/research-reagents/single-color-antibodies-ruo/bv510-hamster-anti-mouse-cd3e.563024
Mouse anti-Mouse NK-1.1 (PK136) for flow cytometry, https://www.bdbiosciences.com/zh-cn/products/reagents/flow-cytometry-reagents/research-reagents/single-color-antibodies-ruo/bv786-mouse-anti-mouse-nk-1-1.740853
Rat anti-Mouse CD11b(M1/70) for flow cytometry, https://www.bdbiosciences.com/zh-cn/products/reagents/flow-cytometry-reagents/research-reagents/single-color-antibodies-ruo/bv711-rat-anti-cd11b.563168
Rat anti-Mouse Ly-6G Antibody(1A8-Ly6g) for flow cytometry, https://www.thermofisher.cn/cn/zh/antibody/product/Ly-6G-Antibody-clone-1A8-Ly6g-Monoclonal/11-9668-82
Rat anti-mouse F4/80 Antibody(BM8) for flow cytometry, https://www.biolegend.com/en-us/products/pe-anti-mouse-f4-80-antibody-4068
Rat anti-Mouse I-A/I-E(M5/114.15.2) for flow cytometry, https://www.bdbiosciences.com/zh-cn/products/reagents/flow-cytometry-reagents/research-reagents/single-color-antibodies-ruo/bv650-rat-anti-mouse-i-a-i-e.563415
Hamster anti-Mouse CD11c(N418) for flow cytometry, https://www.bdbiosciences.com/zh-cn/products/reagents/flow-cytometry-reagents/research-reagents/single-color-antibodies-ruo/bv605-hamster-anti-mouse-cd11c.744179
Rat anti-mouse Ly-6C Antibody(HK1.4) for flow cytometry, https://www.biolegend.com/en-us/products/brilliant-violet-421-anti-mouse-ly-6c-antibody-8586
Rat anti-mouse/human CD11b antibody(M1/70) for flow cytometry, https://www.biolegend.com/en-us/products/percp-cyanine5-5-anti-mouse-human-cd11b-antibody-4257
Rat anti-mouse F4/80 Antibody(BM8) for flow cytometry, https://www.thermofisher.cn/cn/zh/antibody/product/F4-80-Antibody-clone-BM8-Monoclonal/25-4801-82
Rat anti-mouse CD163 Antibody(TNKUPJ) for flow cytometry, https://www.thermofisher.cn/cn/zh/antibody/product/CD163-Antibody-clone-TNKUPJ-Monoclonal/63-1631-82
Rabbit anti-Murine RELMα Antibody(polyclonal) for flow cytometry, PMID: 28495875, https://www.peprotech.com/zh/biotinylated-anti-murine-relm-2
Mouse anti-Ki-67 Antibody(B56) for flow cytometry, https://www.bdbiosciences.com/zh-cn/products/reagents/flow-cytometry-reagents/research-reagents/single-color-antibodies-ruo/bv421-mouse-anti-ki-67.565929
Rat anti mouse CD68 Antibody(FA-11) for IF, https://www.abcam.cn/cd68-antibody-fa-11-ab53444.html
Rat anti Human/Mouse TREM2 Antibody(237920) for flow cytometry and IF, https://www.rndsystems.com/cn/products/human-mouse-trem2-antibody-237920_mab17291
Goat anti mouse TREM2 Antibody(polyclonal) for IF, https://www.abcam.cn/trem2-antibody-ab95470.html
Mouse anti-mouse CD45.1(A20) for flow cytometry, https://www.bdbiosciences.com/zh-cn/products/reagents/flow-cytometry-reagents/research-reagents/single-color-antibodies-ruo/percp-cy-5-5-mouse-anti-mouse-cd45-1.560580
Mouse anti-mouse CD45.2(104) for flow cytometry, https://www.bdbiosciences.com/zh-cn/products/reagents/flow-cytometry-reagents/research-reagents/single-color-antibodies-ruo/bv605-mouse-anti-mouse-cd45-2.563051
BV421 Mouse IgG1, k Isotype Control (X40) for flow cytometry, https://www.bdbiosciences.com/zh-cn/products/reagents/flow-

cytometry-reagents/research-reagents/flow-cytometry-controls-and-lysates/bv421-mouse-igg1-k-isotype-control.562438
Mouse anti-Mouse CD163 (ED2) for IF, https://www.scbt.com/zh/p/cd163-antibody-ed2
Rabbit anti-Mouse TOMM20 (ERP15581-54) for IF, https://www.abcam.cn/tomm20-antibody-epr15581-54-mitochondrial-marker-ab186735.html
Rabbit anti-Cardiac Troponin I (polyclonal) for IF, https://www.abcam.cn/cardiac-troponin-i-antibody-ab47003.html
Rat anti-Mouse LAMP1 for IF, https://dshb.biology.uiowa.edu/1D4B
Alexa Fluor 594 Donkey anti-Rat antibody for IF, https://www.thermofisher.cn/cn/zh/antibody/product/Donkey-anti-Rat-IgG-H-L-Highly-Cross-Adsorbed-Secondary-Antibody-Polyclonal/A-21209
Alexa Fluor 488 Donkey anti-Mouse antibody for IF, https://www.thermofisher.cn/cn/zh/antibody/product/Donkey-anti-Mouse-IgG-H-L-Highly-Cross-Adsorbed-Secondary-Antibody-Polyclonal/A-21202
Alexa Fluor 594 Goat anti-Mouse antibody for IF, https://www.thermofisher.cn/cn/zh/antibody/product/Goat-anti-Mouse-IgG-H-L-Cross-Adsorbed-Secondary-Antibody-Polyclonal/A-11005
Alexa Fluor 488 Donkey anti-Rabbit antibody for IF and flow cytometry, https://www.thermofisher.cn/cn/zh/antibody/product/Donkey-anti-Rabbit-IgG-H-L-Highly-Cross-Adsorbed-Secondary-Antibody-Polyclonal/A-21206
Alexa Fluor 647 Donkey anti-Rat antibody for IF and flow cytometry, https://www.thermofisher.cn/cn/zh/antibody/product/Donkey-anti-Rat-IgG-H-L-Highly-Cross-Adsorbed-Secondary-Antibody-Polyclonal/A48272

Eukaryotic cell lines

Policy information about cell lines

Cell line source(s): The L929(CCL-1) cell line was obtained from the American Type Culture Collection(ATCC,Rockville.MD,USA).

Authentication: The L929 cell line was authenticated by STR profiling.

Mycoplasma contamination: All cell lines have no mycoplasma contamination.

Commonly misidentified lines (See ICLAC register): No commonly misidentified lines were used.

Animals and other organisms

Policy information about studies involving animals; ARRIVE guidelines recommended for reporting animal research

Laboratory animals: 4-20 weeks old male mice were used. C57BL/6 wild-type (WT) mice were purchased from Shanghai SLAC Laboratory Animal Center. Mice were housed under controlled conditions, namely 22°C, 45–65% relative humidity, and 12:12 light-dark cycle. Professor Marco Colonna (Washington University, St. Louis, USA) kindly provided Trem2-/- mice. Professor Min Shang (Zhejiang University, Hangzhou, China)kindly provided αMHC-Cre mice. The following mice were purchased from the Jackson Laboratory: Rosa26-stop-tdTomato, Cx3cr1-CreERT2–IRES–YFP, and C57BL/6 CD45.1 mice (B6.SJL-Ptprca Pepcb/BoyJ).

Wild animals: Wild animals were not used.

Field-collected samples: No field-collected samples were involved.

Ethics oversight: The animal experiments were approved by the Animal Care and Use Committee of Zhejiang University School of Medicine and performed according to institutional guidelines.

Note that full information on the approval of the study protocol must also be provided in the manuscript.

Flow Cytometry

Plots

Confirm that:

- [x] The axis labels state the marker and fluorochrome used (e.g. CD4-FITC).
- [x] The axis scales are clearly visible. Include numbers along axes only for bottom left plot of group (a 'group' is an analysis of identical markers).
- [x] All plots are contour plots with outliers or pseudocolor plots.
- [x] A numerical value for number of cells or percentage (with statistics) is provided.

Methodology

Sample preparation: Mice were anesthetized by isoflurane inhalation. Afterward, the hearts were perfused with perfusion buffer (1× DPBS with 0.8 mM CaCl2) until complete blanching of the liver (~5 minutes) to remove peripheral blood from the chambers. Subsequently, the hearts were isolated, atria and valves were removed, and ventricles were minced to ~1 mm cubes. The hearts were digested with 0.25 mg/ml of Liberase TL (Sigma-Aldrich), 20 μg/ml Dnase I (Sinopharm Chemical Reagent Co.), and 10 mM HEPES (Sigma-Aldrich) in serum-free DMEM (Gibco) medium at 37°C for 15 minutes. After that, the tissue suspension was triturated using 1000 μL micropipettes. The resulting cell suspension was filtered through a 70 μm filter to remove residual undigested tissue pieces, washed with PBS containing 2% FBS.

Instrument	BD LSRFortessa, Beckman moflo Astrios EQ
Software	BD FACSDiva 8.0.1, FlowJo 10.6.2 (Tree Star Inc, OR, USA)
Cell population abundance	The abundance of immune cells in total heart tissue digest is ~1-2%. Purity was determined by flow cytometry and confirmed in our data analysis.
Gating strategy	Gating strategy is displayed in extended data fig. 2d and extended data fig. 3b.

[x] Tick this box to confirm that a figure exemplifying the gating strategy is provided in the Supplementary Information.
